# A revised Sorensen model: Simulating glycemic and insulinemic response to oral and intra-venous glucose load

**DOI:** 10.1371/journal.pone.0237215

**Published:** 2020-08-14

**Authors:** Simona Panunzi, Marcello Pompa, Alessandro Borri, Vincenzo Piemonte, Andrea De Gaetano

**Affiliations:** 1 Institute of System Analysis and Informatics (IASI) “A. Ruberti”, National Research Council (CNR), Rome, Italy; 2 Unit of Chemical-physics Fundamentals in Chemical Engineering, Department of Engineering, University Campus Bio-Medico di Roma, Rome, Italy; University of Pisa, ITALY

## Abstract

In 1978, Thomas J. Sorensen defended a thesis in chemical engineering at the University of California, Berkeley, where he proposed an extensive model of glucose-insulin control, model which was thereafter widely employed for virtual patient simulation. The original model, and even more so its subsequent implementations by other Authors, presented however a few imprecisions in reporting the correct model equations and parameter values. The goal of the present work is to revise the original Sorensen’s model, to clearly summarize its defining equations, to supplement it with a missing gastrio-intestinal glucose absorption and to make an implementation of the revised model available on-line to the scientific community.

## Introduction

Diabetes is a metabolic disorder that affects millions of people worldwide and depends on the reduced capacity or the complete failure of the pancreas to produce insulin, possibly combined with the resistance of tissues to insulin action. People affected by diabetes, if not properly treated, present elevated levels of glucose in the bloodstream (hyperglycemia), and if not well controlled may develop long-term severe chronic complications such as retinopathy, stroke, nephropathy, neuropathy, or even experience dangerous and potentially lethal episodes of hypoglycemia [1–6].

In this framework, the need for developing tailored treatment approaches has led to the use of mathematical models able to represent the glucose/insulin dynamic response of the single patient, for instance with the objective of fully automating-insulin delivery. With the goal of maintaining glucose levels inside a narrow range of values, a number of closed-loop control methods have been designed, often relying on a limited or sparse number of observations, approach that is often referred to with the general term *artificial pancreas* (see e.g. [7–12] and references therein); these systems base their efficacy, in terms of in-silico testing, on reliable mathematical models of glucose homeostasis.

Over the last sixty years, several efforts have been made for the representation of the glucose/insulin system through the use of compartmental models, some simpler (“minimal models”), with few equations and parameters [13–16], others more complex, including interactions among many players (insulin, glucagon, free fatty acids‥). Among the most widely used comprehensive multi-compartment models of the glucose-insulin system there are the UVa-Padova model [17–20], the Hovorka model [21, 22] and the model developed by Sorensen [23]. In particular, the Sorensen model is perhaps the most complex among these, incorporating 22 differential equations (mostly nonlinear), representing glucose concentrations in the brain, heart and lungs, liver, gut, kidney and periphery, with about 135 parameters (including the initial conditions of the state variables). The values of the many model parameters were decided on the basis of a careful literature research. Many researchers subsequently based their work on the Sorensen model ([24–39] represent just an example), but because of its complexity only few authors used it for the development of control algorithms [31, 35, 38, 39]. The model delivers a detailed and well-documented representation of the physiological mechanisms, but, possibly due to its complexity, several scientists who employed and implemented it did not perform a sufficiently thorough, painstaking analysis of the model itself, thereby inheriting some errors from the original Sorensen presentation.

Moreover, while intravenous glucose administration experiments and continuous glucose infusion rate experiments can be explicitly represented by the original model, oral glucose administration lacks such an explicit representation. In fact, the 100g oral glucose test described by the Author in his original work is simulated by means of the direct introduction of a gut glucose absorption rate term in the gut mass balance equation (*r*_*oga*_), bypassing the glucose pathway from stomach to gut and adopting an empirical functional formulation for the time curve of the absorption rate. In the process of adapting the model to experimental data, the Author adjusted the rate of gut glucose absorption in order to match observed blood glucose concentrations with model-predicted peripheral blood glucose concentrations.

Another limitation of the Sorensen model is the inability of the pancreas sub-model to appropriately secrete insulin in response to an oral glucose load, which would have required some description of the action of incretin hormones. This limitation was bypassed by the Author by empirically estimating the required rate of pancreatic insulin output to adjust predicted insulin concentrations with respect to observed insulin experimental data [23].

The objective of the present work is twofold: first, we want to highlight and correct the errors appearing in the original version of the Sorensen’s model, to implement a carefully revised version of the model, and to make the implementation available on-line to the scientific community. The implementation is provided both in user-to-machine and machine-to-machine versions at the address: http://biomatlab.iasi.cnr.it/models/login.php (access as a Guest).

Matlab code is also downloadable from the same link.

The second objective is to enrich the model by introducing a description of the gastrointestinal tract (to simulate alimentary glucose intake, digestion and absorption), exploiting a previously published glucose absorption formulation [40], which was demonstrated to adapt well to experimental data from individuals ranging from normal subjects to type-2 diabetic patients.

## Methods

### Sorensen model analysis

**Fig 1:**
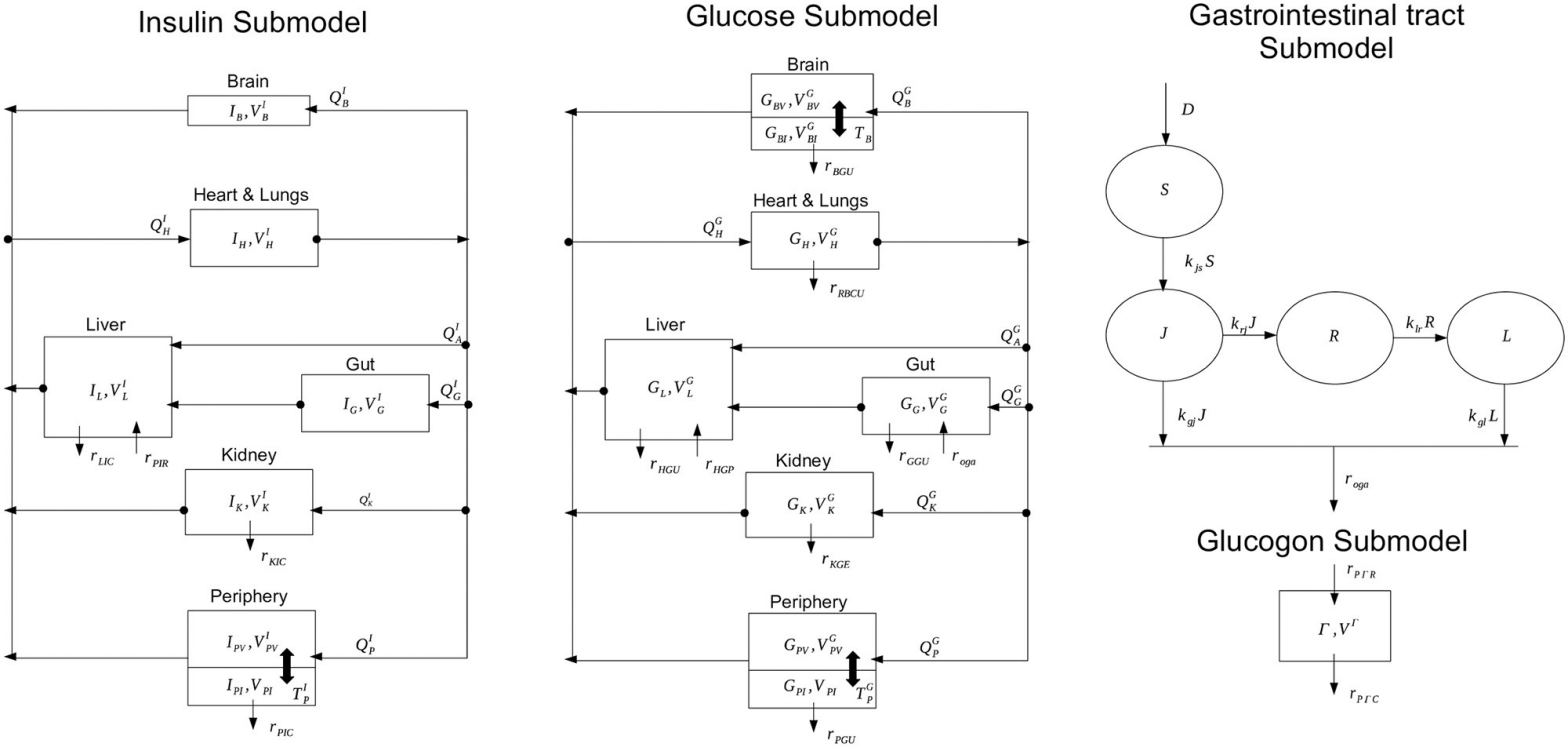
Schematic representation of the Sorensen model (block diagrams). With arrows representing flows between compartments, and block diagram of the gastric intestinal tract. Output from the gastric intestinal model represents the input into the *G*_*G*_ compartment; arrows represent mass transfer.

The Sorensen model [23] is one of the most complex physiological models describing glucose homeostasis. It has been vastly cited and used to simulate virtual patients with the aim of validating control algorithms in the framework of artificial pancreas development. It consists of three sub-models describing the glucose concentration time-course in brain, liver, heart and lungs, periphery (tissue and muscles), gut and kidney and includes the pancreatic release of insulin and glucagon. A scheme of the model is reported in Fig 1.

The original model is represented by the first two block diagrams; the third diagram represents instead the added gastrointestinal tract, whose output constitutes one of the inputs into the *G*_*G*_ Sorensen compartment. Appendix A1 reports the complete and corrected Sorensen model along with all the parameter values and the initial conditions of the equations. In presenting the model, Sorensen reported detailed reasoning and literature references for each equation and parameter value adopted, justifying each single choice with an accurate and broad discussion of the involved physiological aspects. A summary of the final model formulation was reported at page 213 of his dissertation in the subsection “Summary of Model Equations, Parameter Values, and Mathematical Nomenclature”. Given its complexity, most of the researchers who used the model in their activity referred this summary section without double-checking the equations reported in the other sections of the original work. Some other Authors then referred to previous publications based on the Sorensen model, thereby inheriting the same imprecisions from their sources. Table 1 reports a list of errors we found in the original dissertation and which were incorporated in later work using the model [26, 31, 35, 38, 39].

**Table 1:** Sorensen imprecisions and the relative corrections.

Sorensen Summary and Initial Condition	Page	Correct form	ID
$r_{KGE}(\frac{mg}{min})=71+71tanh[0.11(G_{K}-460)]$	216	$r_{KGE}(\frac{mg}{min})=71+71tanh[0.011(G_{K}-460)]$	(A)
$0<G_{K}<460\frac{mg}{min}$	216	$0<G_{K}<460\frac{mg}{dL}$	(B)
$r_{KIC}=F_{KIC}\left[Q_{K}^{I}I_{K}\right]$	219	$r_{KIC}=F_{KIC}\left[Q_{K}^{I}I_{H}\right]$	(C)
$\frac{dQ}{dt}=k(Q-Q_{0})+\gamma P-S$	219	$\frac{dQ}{dt}=k(Q_{0}-Q)+\gamma P-S$	(D)
$G_{PI}=G_{PV}-\frac{r_{BGU}}{V_{PI}}T_{P}^{G}$	264	$G_{PI}=G_{PV}-\frac{r_{PGU}}{V_{PI}}T_{P}^{G}$	(E)

Model simulations highlight the fact that the identified errors produce a clearly different model behavior: kidney glucose excretion appears to be slower (A), adopted initial conditions do not appear to be at equilibrium (C and E), insulin secretion appears to be incorrect (D). Error C was present for example in [26], [35], [38], [39] and in [31] where reference [35] was used as source. Error A was inherited by [26] and [38]. We hypothesize that the problem highlighted in this last publication, where equilibrium points appear to lie in a “non-feasible region”, could in fact be due to the introduction of the above errors in the Sorensen model implementation used.

### Sorensen re-implementation

#### Implementation and test of the original version

The Sorensen model was implemented by following the internal standard procedures of the CNR-IASI BioMatLab. Implementation of the model equations was conducted following the MoSpec (model specification) approach, an internally developed automatic system, which takes as input a spreadsheet containing all the model specifications (description of the equations, parameter names, initial conditions, parameter values, latex and matlab syntax) from which computational routines in Matlab [41], R [42] and C++ [43] are automatically generated, along with a LaTeX document containing the model equations as well as the program code produced for the implementation. Having automated code development and automatic comparison of the original mathematical formulation with the actual coding implementation allows fast and thorough code verification. In order to compare predictions obtained by Sorensen in his original work and predictions obtained by our system, a number of test cases presented by Sorensen were simulated:

- a standard 0.5 g/kg Intra Venous Glucose Tolerance Test (IVGTT);
- a variable-dose IVGTT comparison (0.05, 0.2, 0.5 and 0.75 g/kg);
- a 0.04 U/kg Intravenous Insulin Tolerance Test IVITT;
- a continuous intravenous insulin infusions (0.25, 0.4 mU/kg).

The next section reports the results obtained.

#### The updated original version: Modelling the oral glucose assumption

The original work of Sorensen presents a series of simulations showing the good adaptation of the model to experimental data. Some of the simulations were those described above and show the time courses of glucose and insulin concentrations when glucose and/or insulin were administered intravenously as a bolus or as a constant infusion.

Sorensen also tested the performance of the model when glucose was administered orally: in this case he was forced to derive empirically both the rate of insulin secretion and the rate of glucose appearance. Two major limitations are indeed present in the Sorensen model: both the incretin effect and the rate of gastric uptake are not explicitly modeled. When glucose is ingested orally, glucose-induced insulin secretion by pancreatic *β*-cells is potentiated with respect to the case in which glucose is administered intravenously. This is due to the incretin effect of at least two hormones, glucose-dependent insulinotropic polypeptide (GIP) and glucagon-like peptide-1 (GLP-1), both released in the gut from enteroendocrine cells when glucose goes through the intestinal tract. This potentiation effect is not explicitly modelled by Sorensen and indeed the set of parameter values provided by Sorensen does not produce an insulin secretion rate compatible with the insulin concentrations that are normally observed during an oral glucose load. In order to obviate this discrepancy, Sorensen bypassed completely the pancreatic insulin release sub-model and iteratively adjusted the rate of assumed pancreatic insulin secretion into the circulating insulin sub-model, until the predicted serum insulin concentrations matched the experimental data. The same procedure was adopted for the rate of gut oral glucose absorption (*r*_*oga*_, mg/min), for which an empirical function was used as input in the glucose gut compartment and then adjusted so as to match the observed glucose concentrations with model predictions.

In the present work, an improved version of the Sorensen model is implemented, incorporating into the original 1978 version a previously published model of the Oral Glucose Tolerance Test, the SIMO model [40]. When choosing which representation of gastrointestinal glucose absorption to use in order to integrate the original Sorensen model, we reviewed several options from the literature. Appendix A2 describes the other gastrointestinal glucose absorption models that we considered. We finally choose the SIMO model due to its relative simplicity, to the fact that it represents mechanistically anatomical compartments and transfers, and to the fact that it fits available observations well. The SIMO model is composed of four compartments corresponding indicatively to Stomach, Jejunum and Ileum, plus a delay compartment between Jejunum and Ileum. Although the original SIMO model explicitly incorporates the incretin mechanism, making insulin secretion depend on the glucose content in the Jejunum and Ileum, the part of the model used here is only that related to the glucose absorption route. In the present formalization, stomach emptying and transfer from stomach to proximal and distal intestinal tract are modelled by linear dynamics. This approach was sufficient in the original SIMO model to obtain a very good adaptation of the model to data from subjects with different degree of glycemic impairment (from normal glucose tolerance to impaired fasting glucose and impaired glucose tolerance up to Type-2 Diabetes Mellitus). The equations inherited from the SIMO model are reported below:

$$\begin{array}{ccc} \frac{dS}{dt}=-k_{js}S, & & S(0)=D \end{array}$$ (1)

$$\begin{array}{ccc} \frac{dJ}{dt}=k_{js}S-k_{gj}J-k_{rj}J, & & J(0)=0 \end{array}$$ (2)

$$\begin{array}{ccc} \frac{dR}{dt}=-k_{lr}R+k_{rj}J, & & R(0)=0 \end{array}$$ (3)

$$\begin{array}{ccc} \frac{dL}{dt}=k_{lr}R-k_{gl}L, & & L(0)=0 \end{array}$$ (4)

$$\begin{array}{ccc} r_{oga}=f(k_{gj}J+k_{gl}L), & & r_{oga}\left(0\right)=0 \end{array}$$ (5)

where D is the orally administered quantity of glucose whereas S, J and L represent the quantity of glucose in the stomach, in the jejunum and in the ileum compartments respectively. R is a delay compartment, necessary to approximate the absorption profile of glucose over time: see [40] and [44] for details. The function *r*_*oga*_ represents the rate of gut oral glucose absorption, which acts as input into the original Sorensen model and which substitutes the empirical test function described above. In other words, the function *r*_*oga*_ constitutes the link between the SIMO model and the Sorensen model (see Fig 1) and is substituted into the Sorensen model equation

$$\begin{array}{c} V_{G}^{G}\frac{dG_{G}}{dt}=Q_{G}^{G}G_{H}-Q_{G}^{G}G_{G}+r_{oga}-r_{GGU}. \end{array}$$ (6)

The incretin effect was not explicitely modelled by Sorensen and the original insulin model and pancreatic insulin release remain unchanged in the present formulation. Most of the parameters related to insulin metabolism are allow to vary freely, in order to describe a pancreatic insulin release able to produce a good fit to observed insulin concentrations. The next section reports the procedure carried out to test the ability of this improved version of the Sorensen model to reproduce an Oral Glucose Tolerance Test.

### Model fitting and sensitivity analysis

The Sorensen insulin pancreatic release sub-model equations are reported below:

$$r_{PIR}=\frac{S(G_{H})}{S(G_{H}^{B})}r_{PIR}^{B}$$ (7)

$$\frac{dP}{dt}=\alpha [P_{\infty }-P]$$ (8)

$$\frac{dI}{dt}=\beta [X-I]$$ (9)

$$\frac{dQ}{dt}=K(Q_{0}-Q)+\gamma P-S$$ (10)

$$S=[M_{1}Y+M_{2}{\left(X-I\right)}^{0^{+}}]Q$$ (11)

$$X=\frac{{\left(G_{H}\right)}^{\beta_{pir1}}}{{\left(\beta_{pir2}\right)}^{\beta_{pir1}}+\beta_{pir3}{\left(G_{H}\right)}^{\beta_{pir4}}}$$ (12)

$$P_{\infty }=Y={\left(X\right)}^{\beta_{pir5}}$$ (13)

$$r_{PIR}^{B}=\frac{Q_{L}^{I}}{1-F_{LIC}}I_{L}^{B}-Q_{G}^{I}I_{G}^{B}-Q_{A}^{I}I_{H}^{B}$$ (14)

The free parameters to be estimated are those reported in Eqs [1–5; 7–14], except for parameter *Q*_0_ which was kept fixed at its original value as identified by Sorensen. For a detailed description of the Sorensen model variables and parameters refer to [23].

The parameter identification process was conducted according to four steps:

**STEP 1** The *r*_*oga*_ time course, as empirically derived by Sorensen, was used as data input: data were graphically extracted (27 data points) and used to estimate the SIMO model parameters, so as to predict a curve that was as close as possible to the original Sorensen *r*_*oga*_ function.
**STEP 2** The SIMO model parameters were then kept fixed and one hundred optimization procedures were performed to estimate the free parameters of the Sorensen pancreatic insulin release sub-model, using as data glucose and insulin concentrations as well as points extracted from the *r*_*PIR*_ time course (25 data points). In each one of the 100 optimization procedures the starting value used for each of the free parameter to be estimated was a random realization from a normal distribution centered on the parameter value reported by Sorensen with a standard deviation equal to 15% of the same parameter value.
**STEP 3** A final optimization procedure was performed by letting all parameters from the above two steps vary freely, using all available data points from the four “observed” state variables (plasma glucose and insulin concentrations, *r*_*oga*_ and *r*_*PIR*_). This last optimization procedure was carried out using as starting value the optimum parameter vector from STEP 1 and the “best” optimum (the one producing the smallest loss function) among the 100 optimization procedures from STEP 2.
  An approximation to the Variance-Covariance matrix of the model parameter vector has been computed as [*J*^*T*^ *S*^−1^ *J*]^−1^, where *J* represents the Jacobian in the obtained optimum and *S* is Variance-Covariance matrix of the observation error vector, assumed to be diagonal (uncorrelated errors) with variances proportional to the square of the mean response:
  $$\begin{array}{ccc} S(i,j) & = & \sigma^{2}f{\left(t_{j},\theta \right)}^{2},i=j\\ S(i,j) & = & 0,i\neq j \end{array}$$ (15)
  where *σ*, the scale parameter, is the coefficient of variation.
**STEP 4** Two hundred optimization procedures were then performed to test model “a-posteriori” identifiability: each of the free parameters of the Sorensen insulin pancreatic release sub-model was initialized perturbing its optimum value from STEP 3: for each parameter to be estimated the new starting value in each of the 200 optimization procedures was sampled from a normal distribution centered on the parameter optimum value, with a standard deviation equal to its 15%.

Each optimization process was performed by minimizing the sum of the weighted squared residuals (weighted least-squares estimation, with weights the inverse of the squared expectations) over 5 free parameters in STEP 1 (Table 2 with parameter *f* kept fixed at 1), 11 free parameters in STEP 2 (Table 3 with parameter *Q*_0_ fixed at its original value) and 16 parameters in STEPs 3 and 4.

**Table 2:** STEP 1 results. Simo model parameters before (as reported in [40]) and after the optimization process for *r*_*oga*_ data fitting.

Parameters [Units]	Before	After
*K*_*js*_ [1/min]	0.25	0.028237
*K*_*gl*_ [1/min]	0.1	0.0180942
*K*_*gj*_ [1/min]	0.042	0.0329673
*K*_*rj*_ [1/min]	0.09	0.0344046
*K*_*lr*_ [1/min]	0.06	0.0513802
*f* [#]	0.7	1

**Table 3:** STEP 3 results. Sorensen pancreatic insulin release model parameters before and after the optimization process.

Parameters [Units]	Before	After
*K*_*js*_ [1/min]	0.028	0.026
*K*_*gl*_ [1/min]	0.018	0.035
*K*_*gj*_ [1/min]	0.033	0.032
*K*_*rj*_ [1/min]	0.034	0.029
*K*_*lr*_ [1/min]	0.051	0.026
*f* [#]	0.7	1
*α* [1/min]	0.0482	0.014
*β* [1/min]	0.931	15.558
*K* [1/min]	0.00794	0.0145
*Q*_0_ [pmol]	44310	44310
*γ* [pmol/min]	4025	2138.76
*M*_1_ [1/min]	0.00747	0.00012
*M*_2_ [1/min]	0.0958	0.2488
*β*_*pir*1_ [#]	3.27	4.164
*β*_*pir*2_ [mM]	7.333	3.776
*β*_*pir*3_ [#]	2.879	1.837
*β*_*pir*4_ [#]	3.02	3.577
*β*_*pir*5_ [#]	1.11	2.876

While it is true that the model incorporates a high number of free parameters to be simultaneously estimated and that this can lead to overfitting problems, STEPs 3 and 4 were executed in order to have reasonable starting values and reasonable allowable values ranges for each parameter (values for parameters can be assessed for “reasonableness” if each parameter has a direct physiological meaning, which is a good motivation for the use of mechanistic models). The results from STEP 4 give indications about both the variability of the parameter estimates and the correlation between each couple of parameter estimates.

### Bayesian a-posteriori identifiability analysis

For parameters with high coefficients of variation, obtained from the estimated asymptotic variance-covariance matrix of the model parameter vector (STEP 3), a bayesian approach was also implemented in order to evaluate their a-posteriori identifiability with an alternative approach to STEP 4, setting all the others parameters at their optimum value. A Monte Carlo Markov Chain (MCMC) approach was followed, using a Metropolis-Hastings algorithm [45] nested into the Gibbs sampling [46–49], making use of samples from the full conditional distributions. The Bayesian model specification starts with the assignment of a distributional form to the observation error vector, along with the specification of a variance-covariance structure. We assume normally distributed errors, with zero mean and the same variance-covariance matrix as the one adopted in the WLS approach:

$$\begin{array}{c} y_{j}=f\left(t_{j},\theta \right)+\epsilon_{j},\;\;\epsilon_{j}∼N\left(0,S\left(j,j\right)\right) \end{array}$$ (16)

where *y*_*j*_ is the observation at time *t*_*j*_, the errors *ϵ*_*j*_, *j* = 1, …, *n* are assumed to be uncorrelated and where, in analogy with Eq 15, *S*(*jj*) = *σ*^2^[*f*(*t*_*j*_, *θ*)]^2^.

An *a-prior* inverse gamma distribution was assumed for the parameter *σ*^2^ (*σ*^2^ ∼ GI($\frac{\nu }{2}$, $\frac{\tau }{2}$)). The parameter *θ* was log-transformed and a prior uniform distribution was assigned to the parameter vector *log*(*θ*): *p*(*log*(*θ*)) ∼ *U*_*D*_, where *D* is the region delimited by the intervals centred on the logarithm of the parameter optimum values and with limits equal to ±100% of the log-transformed values. The parameter vector, object of the bayesian inference, is therefore the parameter *ξ* = (*η*, *σ*^2^) = (*log*(*θ*), *σ*^2^)).

The full conditional distribution for the scale parameter *σ*^2^ is still an inverse gamma:

$$\begin{array}{c} p\left(\sigma^{2}|y,\theta \right)=GI\left(\frac{n+\nu }{2},\frac{1}{2}\left[{\left(y-f\left(t,\theta \right)\right)}^{T}F^{-1}\left(y-f\left(t,\theta \right)\right)+\tau \right]\right) \end{array}$$ (17)

with *y* being the observation vector, *n* the number of total observations and *F* the diagonal matrix with elements *F*(*jj*) = [*f*(*t*_*j*_, *θ*)]^2^.

Due to non-linearity in the regression model, the full conditional distribution of *η* = *log*(*θ*) cannot be calculated explicitly. However it can be written up to a proportionality constant:

$$\begin{array}{c} p\left(\eta |y\right)=|S{|}^{-\frac{1}{2}}exp\left\{-\frac{1}{2}\left[{\left(y-f\left(t,e^{\eta }\right)\right)}^{T}S^{-1}\left(y-f\left(t,e^{\eta }\right)\right)\right]\right)\}U_{D}\left(\eta \right) \end{array}$$ (18)

The Metropolis-Hastings algorithm was used to obtain samples from the full conditional distribution of parameter the vector *η* = *log*(*θ*). At each iteration of the MCMC, a multinormal distribution for *η* was used as proposal distribution. Mean and variance-covariance matrix of the distribution were derived from the following considerations: using the first order Taylor expansion of the logarithm function, we can approximate *log*(*x*) in a small neighborhood of *x*, *E*(*x*) = *x*_0_:

$${log\left(x\right)|}_{x_{0}}≊log\left(x_{0}\right)+\frac{1}{x}{|}_{x_{o}}\left(x-x_{0}\right)$$ (19)

$$E(log(x))=log(x_{0})$$ (20)

$$Var(log(x))=\frac{1}{x^{2}}{|}_{x_{0}}Var\left(x\right)={\left[CV\left(x\right)\right]}^{2}$$ (21)

With the appropriate substitutions, the proposal multinormal distribution was assumed to be centred on the logarithm of the optimum values from the WLS procedure of STEP 3, while the variance-covariance matrix of *η* was derived from the coefficients of variation computed from the estimated asymptotic variance covariance matrix *σ*^2^[*J*^*T*^ *S*^−1^ *J*]^−1^. While the variance-covariance matrix remained unchanged during the MCMC algorithm, at each iteration of the algorithm the proposal distribution was centred on the values obtained from the preceding iteration. The MCMC algorithm was implemented by building nine independent chains of 10,000 samples, each one beginning from a different parameter starting point. The nine points were chosen as the four vertices of the region D plus the mid points of the edges and the central point of D. For each chain the first 1000 realizations were considered as “burn-in” to let the Markov chains to reach equilibrium and get sufficiently close to the stationary distribution. The characteristics of the empirical distribution of parameter *θ* can be derived from the characteristics of the empirical distribution of *η*. For each couple of parameters, a Credibility Region (CR) can be built from their bivariate empirical distribution: from a 20 × 20 bi-dimensional frequency histogram (400 *cells* in total with relative frequencies *f*_*i*,*j*_), the frequency level ℓ such that the sum of the frequencies for which *f*_*i*,*j*_ > *ℓ* is equal to 95%, is found iteratively:

$$\begin{array}{c} CR_{MCMC}\left(95\%\right)=\left\{cell_{i,j}:f_{i,j}>\ell ,\sum_{i,j}f_{i,j}=0.95\right\} \end{array}$$ (22)

## Results

**Fig 2:**
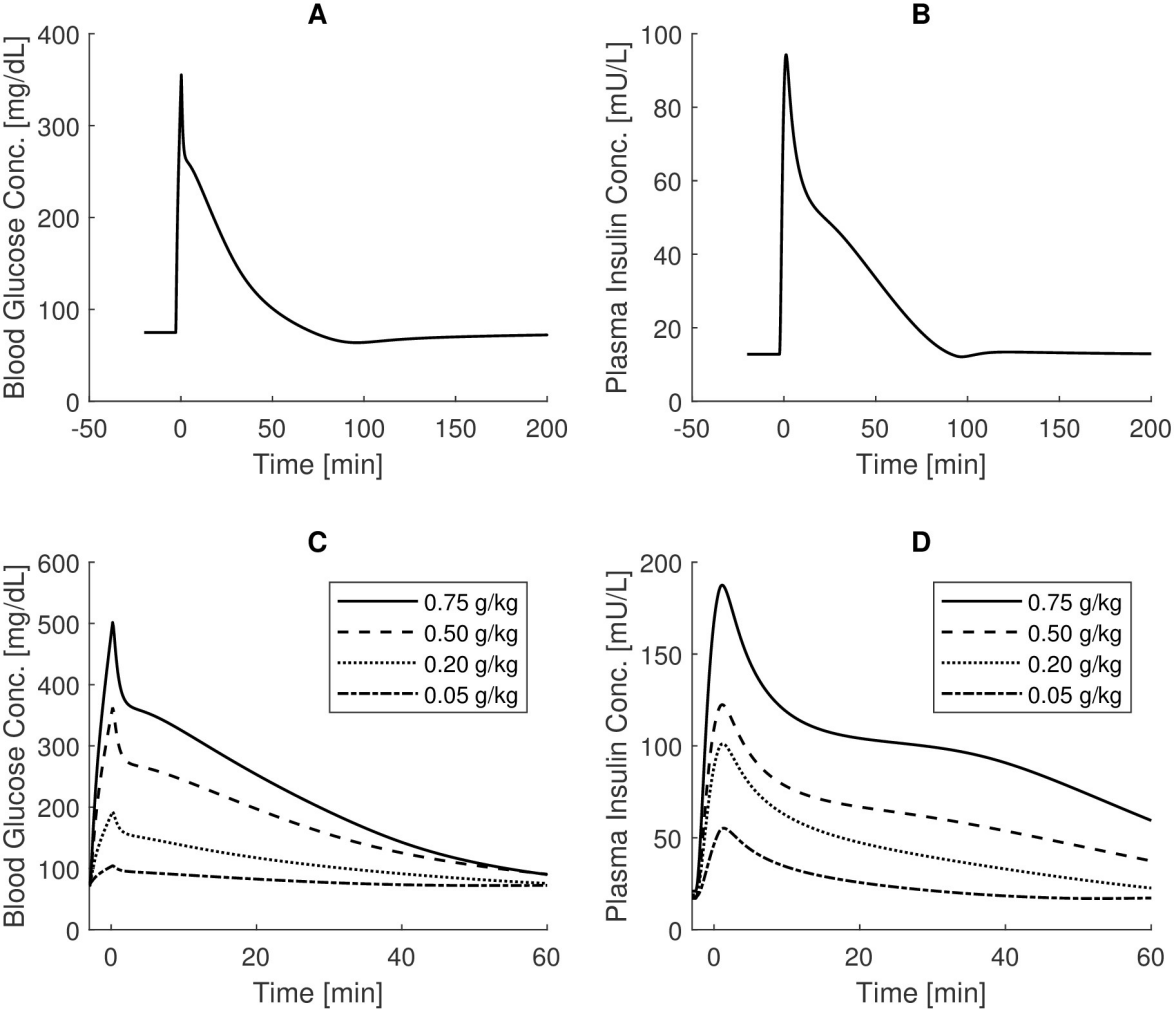
IVGTT Sorensen simulations. Top panels: blood glucose and plasma insulin concentrations over time as derived from the CNR-IASI BiomatLab Sorensen model implementation following a standard 0.5g/kg IVGTT experiment (comparison with Fig 71, page 270 of the original work [23]); bottom panels: blood glucose and plasma insulin concentrations over time obtained in correspondence of variable IVGTT doses (comparison with Fig 73, page 273).

Fig 2, top panels, shows blood glucose and plasma insulin concentrations over time as produced by the CNR-IASI BioMatLab Sorensen model implementation, following a standard 0.5g/kg IVGTT experiment. These curves should be compared with the graphs reported in Fig 71 at page 270 of the original work [23].

Note that peripheral venous blood glucose concentrations were obtained by multiplying by 0.84 the model state variable “peripheral vascular blood water space glucose concentration”, following the indication of the Author. The curves obtained closely resemble the original curves: small deviations, less than 0.20 mg/dl and 10mU/L occur for the maximal concentrations of glucose and insulin respectively. From a qualitative point of view, the simulated curves are perfectly comparable with the original ones.

The bottom panels of Fig 2 show the time courses obtained when simulating the administration of variable IVGTT doses. In this experiment, starting glucose and insulin concentrations were set to different values in correspondence of the different IVGTT doses (table 32 at page 275) and were also different from the values used in the experiment reported in top panels of Fig 2 (89 mg/dl and 12.8 mU/l for glucose and insulin basal concentrations respectively).

Deviations from the original time courses (Fig 73 at page 273 of [23]) are evident only for the insulin concentration profile, which is estimated to be about 20 mU/L lower for the 0.5g/kg IVGTT experiment.

**Fig 3:**
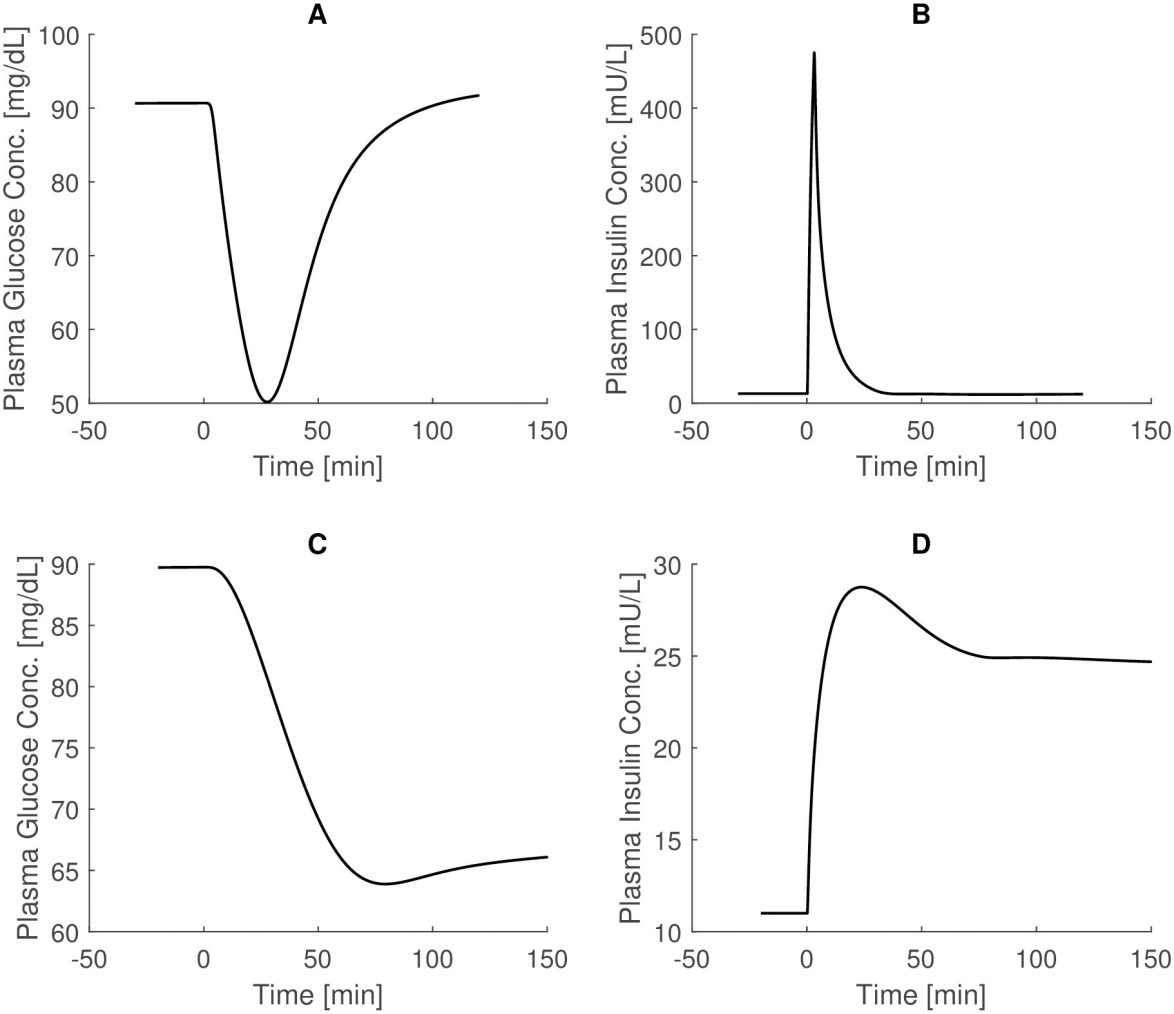
IVITT Sorensen simulations. Top panels: glucose and insulin trends following a 0.04 U/Kg Intravenous Insulin Tolerance Test (IVITT) experiment, with insulin administration in 3 minute (comparison with Fig 74, page 277 of [23]). Bottom panels: glucose and insulin trends following the 150 minutes of 0.25 mU/kg Continuous Intravenous Insulin Infusion experiment (comparison with Fig 75, page 279 of [23]). The glucose state variable is multiplied by 0.925 to obtain the venous plasma glucose concentration.

Fig 3 in the top panels shows the glucose and insulin trends following a 0.04 U/Kg Intravenous Insulin Tolerance Test (IVITT) experiment, with insulin administered over 3 minutes. Reference time courses are shown in Fig 74 at page 277 of [23].

Here the glucose state variable is multiplied by 0.925 to obtain venous plasma glucose concentration, according to the prescriptions of the Author. The curves obtained again closely resemble, both quantitatively and qualitatively, the results by Sorensen.

Results related to the Continuous Intravenous Insulin Infusions experiment are reported in the bottom panel of Fig 3. In this last experiment, 0.25 mU/kg of insulin is infused intravenously for 150 minutes. Also in this case the state variable *G*_*PV*_ (Glucose Periphery Vascular blood water space) is multiplied by 0.925 to obtained venous plasma glucose concentrations. From a comparison with Fig 75 at page 279 of [23], a very good superimposition of the obtained curves with the original ones is apparent.

Table 2 reports the results derived from the first step of the identification process. Values in the second column are the average values reported in [40], which represent the starting point for the optimization process; values in the third column are those obtained after fitting the variables of the SIMO model onto the data extracted from the Sorensen *r*_*oga*_ function.

**Fig 4:**
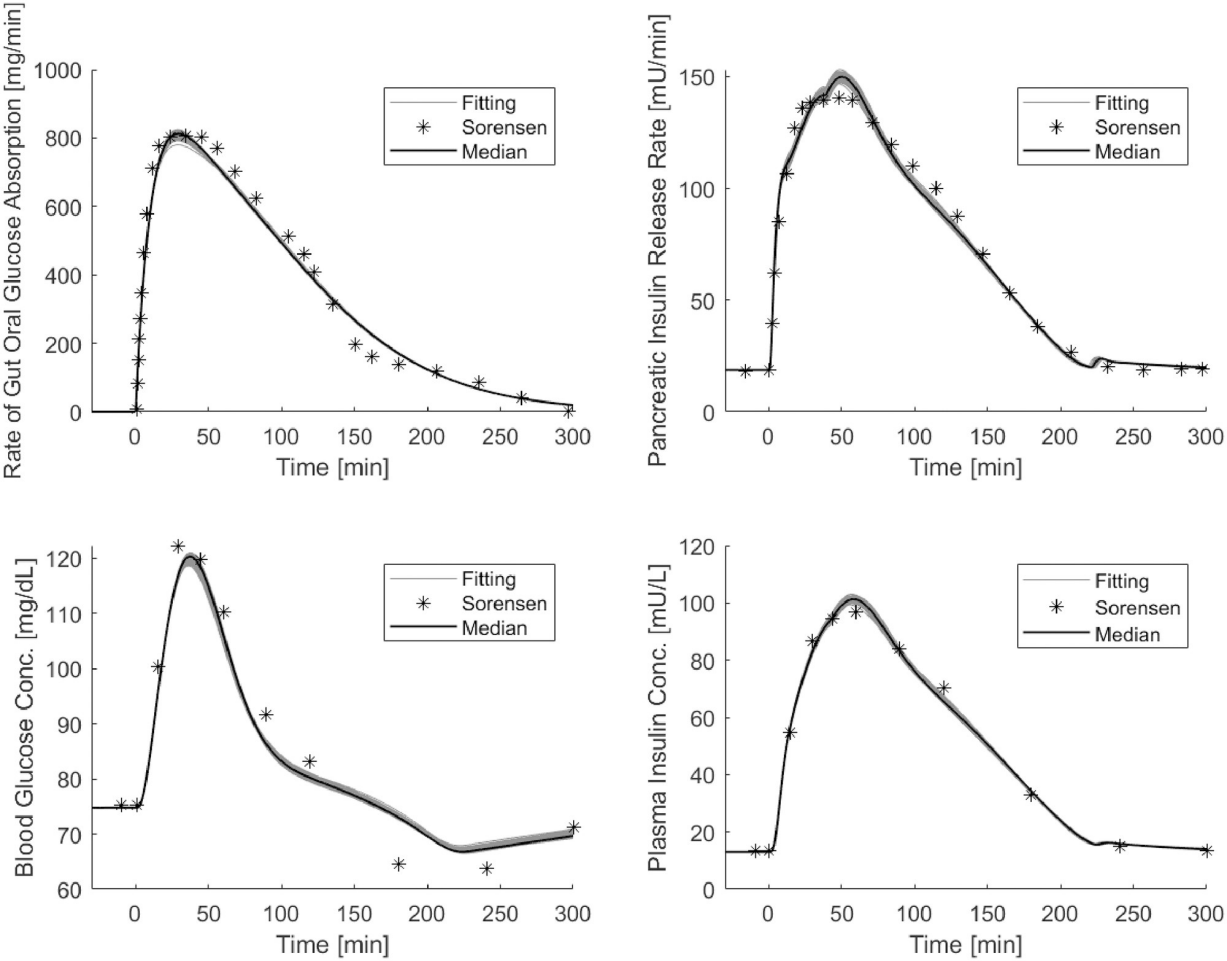
OGTT Sorensen simulations. Panels show the median and the set of predicted curves obtained by the 200 optimization procedures of STEP 4 starting from optimum of STEP 3: top panel on the left reports “observed” (asterisks) data of the *r*_*oga*_ function and the SIMO model predicted rate of appearance (continuous line); top panel on the right reports the “observed” (asterisks) and predicted values (continuous line) of the pancreatic insulin release; bottom panels report observed and predicted values of peripheral glucose (on the left) and insulin (on the right) concentrations.

Fig 4 shows the median and the set of predicted curves obtained from the 200 optimization procedures of STEP 4 starting from optimum of STEP 3.

**Fig 5:**
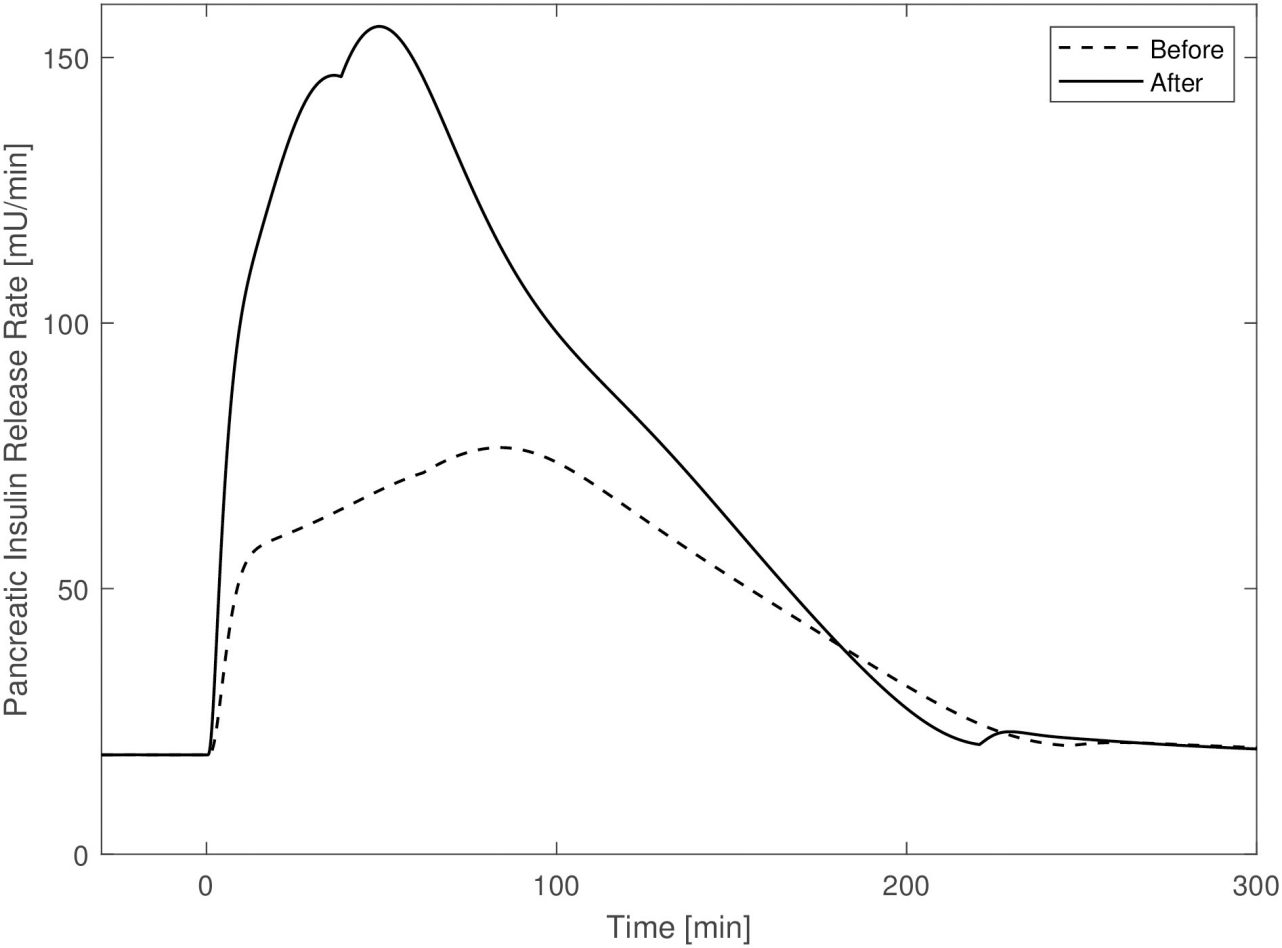
Pancreatic insulin release rate before against after. Pancreatic insulin release rate time courses obtained with parameters set at their original values (dashed line) and with parameter values from STEP 3 (continuous line).

The top panel on the left reports the “observed” data and the predicted rate of appearance, showing the good adaptation of the SIMO model to the *r*_*oga*_ values. Top panel on the right reports the “observed” (asterisks) and predicted values (continuous line) of pancreatic insulin release. The bottom panels report observed and predicted values of peripheral glucose (on the left) and insulin concentrations (on the right). The obtained predictions are very concentrated around their median curve and show a good adaptation of the model to data points. Table 3 reports the insulin release sub-model parameters of the new version of the Sorensen model (including the gastrointestinal tract) before (original values as reported in [23]) and after the optimization procedure from STEP 3. Estimates of many of the insulin secretion sub-model parameters differ from their original values, with a minimal difference of about 18.44% (*β*_*pir*4_) and a maximal difference of about 159.7% (*M*_2_). Fig 5 shows the time courses of the pancreatic insulin release rate obtained from an OGTT experiment with parameters set at their original values (column titled “before” in Table 3) and with the parameter values from STEP 3 (dashed line and continuous line respectively).

Table 4 summarizes the 200 optimization procedures performed to check model identifiability: it reports the mean, standard deviation and coefficient of variations (CVs) of the parameter estimates obtained in the 200 procedures. CVs range from a minimum of 3.9% to a maximum of 22.2%.

**Table 4:** STEP 4 results. Summary of the parameter estimate distributions and parameter standard deviations computed by approximation.

Parameters [Units]	Mean	SD^1^	CV^2^	SD^1^^,^^3^	CV^2^^,^^3^
*K*_*js*_ [1/min]	0.026	0.003	11.8	1.99E-05	0.076
*K*_*gl*_ [1/min]	0.027	0.004	15.8	9.93-05	0.284
*K*_*gj*_ [1/min]	0.036	0.004	10.7	8.48E-05	0.265
*K*_*rj*_ [1/min]	0.033	0.002	6.3	1.09E-04	0.375
*K*_*lr*_ [1/min]	0.030	0.004	14.4	4.79E-05	0.184
*α* [1/min]	0.015	0.002	15.1	1.26E-04	0.90
*β* [1/min]	16.97	2.03	12.0	36.6	235.48
*K* [1/min]	0.015	0.002	14.4	2.14E-04	1.48
*γ* [pmol/min]	2569.6	522.2	20.3	32.5	1.52
*M*_1_ [1/min]	0.00015	3.13E-05	20.8	6.73E-07	0.561
*M*_2_ [1/min]	0.289	0.053	18.2	0.123	49.4
*β*_*pir*1_ [#]	5.148	0.777	15.1	0.094	2.25
*β*_*pir*2_ [mM]	3.746	0.146	3.9	0.017	0.453
*β*_*pir*3_ [#]	2.452	0.545	22.2	0.098	5.33
*β*_*pir*4_ [#]	4.477	0.717	16.0	0.072	2.02
*β*_*pir*5_ [#]	3.066	0.415	13.5	0.023	0.814

^1^ SD: standard Deviation.

^2^ CV: Percent Coefficient of Variation.

^3^ Calculated by ${\hat{\sigma }}^{2}$ [*J*^*T*^ *S*^−1^ *J*]^−1^.

**Fig 6:**
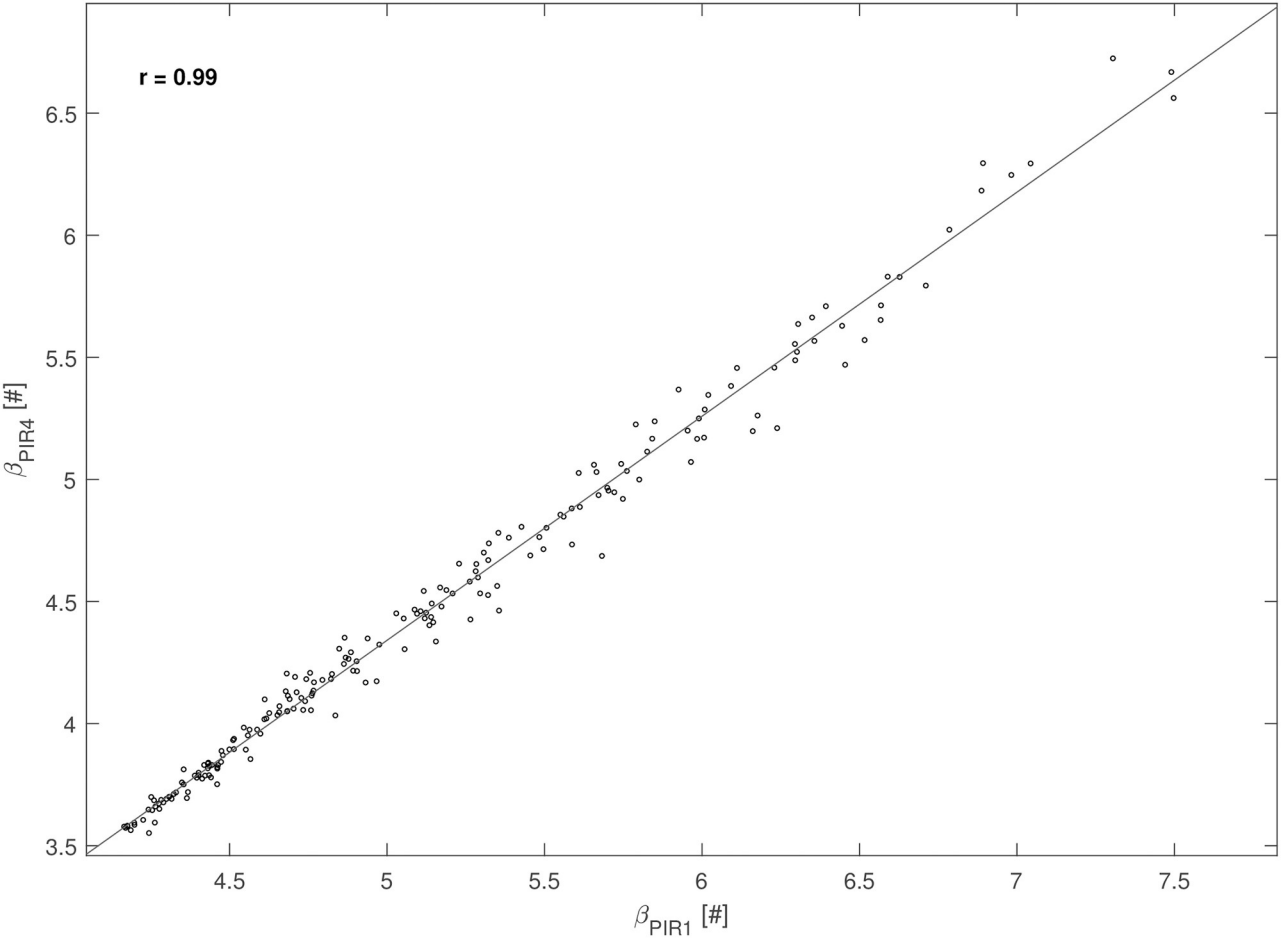
Correlation between Sorensen parameters. Scatter plot of the estimated values of parameters *β*_*pir*1_ and *β*_*pir*4_ obtained in the 200 optimization procedures from STEP 4.

Correlation coefficients (*r*) between each couple of parameters were computed from the 200 optimization procedures to assess if parameters can be uniquely estimated. Many pairs of parameters showed significant correlation: the most correlated pairs (|*r*| ≥ 0.5) were *β*_*pir*1_ and *β*_*pir*3_ (*r* = 0.83); *β*_*pir*1_ and *β*_*pir*4_ (*r* = 0.99); *β*_*pir*3_ and *β*_*pir*4_ (*r* = 0.76); *β*_*pir*2_ and *β*_*pir*5_ (*r* = -0.76); *β*_*pir*2_ and *M*_2_ (*r* = -0.61); *β*_*pir*5_ and *M*_2_ (*r* = 0.54), *P* < 0.0001 for all these correlations. Fig 6 reports the scatter diagram of the 200 estimated pairs (*β*_*pir*1_, *β*_*pir*4_).

**Fig 7:**
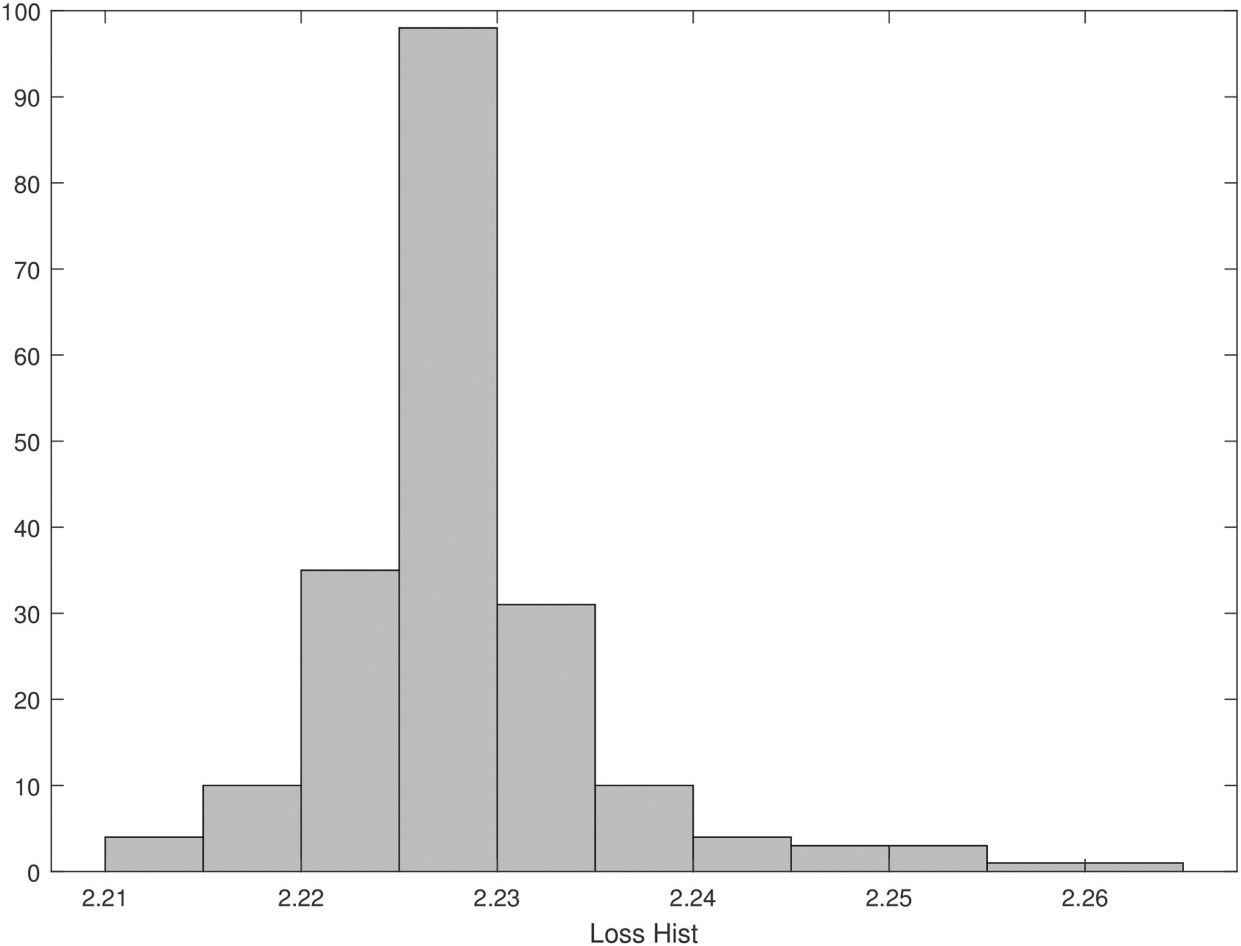
Loss function. Distribution of the loss function values obtained from the 200 optimization procedures of STEP 4.

The minimum and maximum values of the loss function obtained in the 200 optimization procedures were 2.21 and 2.26 respectively, with an average value of 2.23 (SD = 0.007). Fig 7 shows the distribution of the loss function.

**Fig 8:**
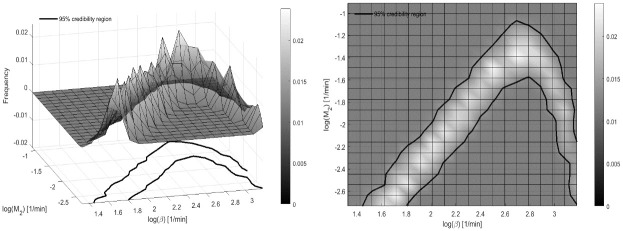
MCMC a-posteriori distribution. Empirical a-posteriori bivariate distribution for parameter (log(*β*), log(*M*_2_)), along with the 95% Credibility Region (CR).

CVs obtained from the approximated Variance-Covariance matrix resulted to be generally lower, except for parameters *β* and *M*_2_ as shown in Table 4. For these two parameters a Bayesian *a-posteriori* identifiability analysis was also performed. The empirical *a-posteriori* bivariate distribution of the logarithms of the two parameters is reported in Fig 8. The figure shows also the empirical Credibility Region (CR). The mean values of the two parameters from the 81,000 realizations of the nine chains are 2.38 and −1.93, for *log*(*β*) and *log*(*M*_2_) respectively, which correspond to a value of 10.79 for *β* and to a value of 0.15 for *M*_2_.

## Discussion

Much previous work addressed the need of developing glucose/insulin models, with different levels of complexity, for a variety of reasons, such as the study of insulin sensitivity or for controlled automatic insulin delivery (artificial pancreas) [7–12]. These models were to be identified on each single patient and had therefore to incorporate the relevant physiological mechanisms in a simplified fashion. More extended models, including a variety of interactions, are in principle more representative of the physiology, and have potentially a greater predictive ability. Such extended models, however, include a large number of parameters: most of them must be fixed to values taken from the literature in order for the remaining ones to be estimated from patient data.

As an example, the development of robust control algorithms for automatic glucose control, capable of maintaining glycemia within a normal range under different perturbations while minimizing the risk of dangerous hypoglicemic episodes, may make good use of adequate, realistic models. Motivated, among other things, by the need to provide plausible virtual diabetic patients for the development of efficient automatic glucose control laws, several complex, “extended” mathematical models of the glucose-insulin system have appeared in the last few years ([17, 20, 23, 26]).

The Sorensen model ([23]) is perhaps the most detailed of these, in terms of documented parameter values and detailed physiological mechanisms. With its 22 nonlinear differential equations and 135 parameters it has been vastly used in glucose control research. This model represents the time course of glucose concentrations in brain, liver, heart & lungs, periphery (tissue and muscles), gut and kidney, and includes submodels for the pancreatic release of insulin and glucagon.

A more recent model, also belonging to the category of extended models, is the UVa/Padova Type 1 Diabetes (T1D) Simulator ([17–20]). Its last version incorporates some time-varying parameters, such as an insulin sensitivity function that changes in order to account for daily tissue glucose uptake variability or for the dawn phenomenon (observed elevated glucose concentrations in the very early hours of the morning). The UVa/Padova Simulator, in almost all of its several subsequent formulations, includes a glucose subsystem, a glucose rate of appearance subsystem, different routes for administered insulin, and a submodel for the glucagon kinetics and secretion.

The Hovorka model ([21]), while substantially simpler than either the Sorensen or the UVa/Padova model, has also been used by several Authors for “closing the patient loop” in the development of automatic control laws [22, 50–52].

The rationale for using the Sorensen model, in particular for using an updated and corrected version of it, resides in the fact that it is a detailed, extended model for the simulation of a virtual patient, that it is well documented (in terms of model equations and parameter values), and that is relatively easy to use it to replicate a wide range of experimental scenarios, as reported in Sorensen’s original work.

Conversely, the UVa/Padova Simulator, which has been commercially distributed, is relatively difficult to implement independently because of the difficulty in finding what parameter values were used by the Authors in their simulations, and also because of the fact that some model equations are only partially described. The UVa/Padova is a simulator for Type I Diabetes Mellitus Patients, and for this reason it lacks any functional representation of the insulin secretion mechanism. In contrast, the Sorensen model allows the simulation of normal subjects, of type 2 Diabetes patients who still maintain some level of endogenous insulin secretion, and of Type I Diabetes patients for which endogenous insulin production is completely replaced by external inputs.

The Sorensen model however presents two major limitations: on one hand it lacks any representation of oral glucose administration and of the corresponding glucose rate of appearance in plasma; a second limitation is that insulin administration is foreseen only by means of intravenous boli or infusions. This latter is an important shortcoming: in clinical practice, subcutaneous delivery represents the normal route of insulin administration, by means of either subcutaneous boli or of wearable pumps. The subcutaneous insulin administration route could be introduced into the model as a further improvement.

In this work we begin with a thorough revision of the Sorensen model, obtaining a model implementation without the several imprecisions reported in the original work and inherited by the Authors who have used this model in their research activity. This corrected model implementation is made available to the scientific community in user-to-machine and machine-to-machine versions at the address: http://biomatlab.iasi.cnr.it/models/login.php (access as a Guest).

Matlab code is also downloadable from the same link.

Moreover, with the aim of making the model more useful for researchers, we propose an improved version supplemented with a mathematical representation of gastrointestinal glucose absorption during oral administration. This updated version of the Sorensen model can also be found at above mentioned address. Following internal BioMatLab standards, the two model versions have been automatically implemented in Matlab, R and C++ starting from formal detailed instructions collected in a Model Specification (MoSpec) spreadsheet. The production documentation includes side-by-side computational code and mathematical description of the model equations, thus allowing a direct check of the correspondence of the computation with the intended mathematical formulation. In the final configuration, Matlab or R user interfaces (with good graphical capabilities and a potentially vast set of ready-made functions for post processing) exploit an underlying compiled fast C++ numerical integration engine. The compiled C++ engine also supports a Visualizer environment, where the user can quickly explore model behaviour corresponding to parameter changes.

The implementations in different languages and the Visualizer environment represent, collectively, a robust approach to verify the correctness of model implementation, which is the BioMatLab standard for model description, implementation and verification.

A series of simulations presented by Sorensen were replicated with our system in order to compare the two implementations. Simulations performed with the corrected version of the Sorensen model closely resemble the curves produced by the original model (see Figs 2 and 3), with very small deviations from what was reported in his work [23]. Small divergences are due to differences in some parameter values: some parameters used by Sorensen (such as basal glycemia or insulinemia) were said to derive from observed population average values but the actual numerical values adopted in the simulations were not reported.

In order to test the ability of the model, supplemented with the gastrointestinal tract submodel, to reproduce insulinemia and glycemia time courses from Oral Glucose Tolerance Tests (OGTTs), the improved version of the Sorensen model was tested on a set of OGTT data already presented by the Sorensen [23]. Fitting the improved model onto OGTT data shows a good qualitative behavior of the model solution with a good adaptation of the curves to experimental points (see Fig 4). We believe that two reasons are responsible for these good results: the first reason is that the Sorensen model, although built on a knowledge basis dating from the Eighties, is a well researched model, incorporating fundamental physiological mechanisms with reasonable mathematical representations. The second reason is that the added gastrointestinal model was taken from the SIMO publication ([40]), where it was demonstrated that linear stomach emptying and linear glucose transfer to the intestine were sufficient to obtain a good representation of glycemia and insulinemia time courses data from subjects with different degree of glycemic impairment undergoing OGTT’s [40].

Adapting the improved model to OGTT data required appropriate changes of some key parameter values of the Sorensen sub-model for insulin secretion. These changes were due to the fact that the Sorensen model does not include explicit modelling of the incretin effect [23]. Modifications of some parameter values however can compensate for this deficiency. It is well known ([53–57]), in fact, that when administering glucose orally, the release of insulin by the pancreas is enhanced with respect to the same amount of glucose administered intravenously. This effect is due to the release of incretin hormones (GIP, GLP1) by the gut when it comes in contact with intraluminal glucose. The Sorensen insulin secretion sub-model does not contemplate an incretin effect, therefore, when using this model with its original parameter values, the model forecast of the insulin levels following oral glucose administration is lower than appropriate. On the other hand, since the glycemic increase after oral administration is slower than after intravenous administration, expected insulin release is also slower. In this case, a better approximation to the actual insulin levels can be obtained by allowing some of the parameter values to change; Table 3 reports the original parameter values and the values obtained after fitting the improved model onto OGTT data. For example, the parameter *γ* (Eq 10) which regulates glucose-driven transfer of insulin to the labile compartment is here estimated, in the case of an OGTT, to roughly half of the original value provided by Sorensen. This result is expected, and depends on the fact that when glucose is infused intravenously its absorption into the bloodstream occurs more rapidly, producing higher peak concentrations than when it is given orally. On the other hand, while *γ* is reduced, producing a slower increase of the releasable insulin Q, parameters *β*_*pir*5_ and *β*_*pir*1_ (Eqs 12 and 13 related to glucose driven early insulin release and to the potentiator factor respectively) are increased, producing a higher total release of the hormone.

The changes made to the parameter values determine changes in the dependent variables. For example, the total amount of labile insulin provision resulted to be increased (since for example *β*_*pir*5_ was increased). Changes in parameter *β*_*pir*1_ also determined large changes in glucose-enhanced early insulin release, *X*(*G*)): since the relationship is highly non-linear, an increment of about 30% of *β*_*pir*1_ results in a large increase of *X*(*G*). A higher release of insulin, in turn, requires a higher insulin inhibition in order to avoid hypoglycemias; this is represented in Sorensen’s model by the dynamics of the inhibitor *I* (Eq 9), which is made to follow the insulin release *X* much faster by increasing the value of the parameter *β*.

The insulin secretion function (Eq 11) is governed by two terms: one acting at the early stage (*M*_2_(*X* − *I*)) and one acting at a later stage (*M*_1_ *Y*). Optimization produced a smaller estimate for *M*_1_ resulting in the function *Y* (the secretory effect of glucose on late insulin release, Eq 13) increasing more slowly for small values of *G*_*H*_ (which occur at the beginning of the experiment), and more quickly when *G*_*H*_ reaches higher concentrations (which, in OGTT’s are maintained for a longer time in comparison with IVGTT’s, requiring therefore an enhanced insulin production; see Eq 12). The reduction in the *M*_1_ parameter may be necessary in order to balance the large increase in Y determined by the increase in *β*_*pir*5_, particularly at high glycemias (at low glycemias the effects of changes in *M*_1_ are negligible).

Moreover, a decrement of the parameter *α* in Eq 8, which represents the speed with which the potentiator factor P moves towards its target *P*_∞_, contributes to maintain high level of insulin at later times.

Since the Sorensen model is very complex, even more so with the proposed addition of a gastrointestinal absorption submodel, it clearly presents identifiability problems. In fact, repeated fitting has shown that some parameter estimates are strongly correlated. In particular, parameters *β*_*pir*1_ and *β*_*pir*4_ present with a correlation coefficient of 0.99 (see Fig 6). Fig 7 shows that the empirical distribution of the loss function values is very concentrated, indicating that different combinations of parameter values produce essentially comparable model predictions and loss function values.

This phenomenon is evident also by observing the results of the bayesian *a-posteriori* identifiability procedure, where the *a-posteriori* distribution of the two parameters that exhibit the largest coefficients of variation (*β* and *M*_2_) is empirically derived. The 95% Credibility Region shows that in correspondence of small values of the parameter *M*_2_, the parameter *β* assumes indistinguishably small or large values. This is likely due to the fact that the action of the insulin inhibitor (which is expressed through the *β* parameter) exerts little influence for low insulin secretion (low values of parameter *M*_2_), in particular during the initial stages of the experiments, where *M*_2_ is most relevant, and where glucose concentrations have not yet reached high levels. Conversely, its contribution becomes more and more important as the secretion increases, so that an increase in *M*_2_ makes an increase of *β* necessary. The descendent tract of the horseshoe corresponds to large values of *β*, when the Inhibitor equals the insulin release *X*(*G*) and the parameter *M*_2_ has little influence (see Eq 11) and may take any value. We can see therefore how the bayesian approach helped to better identify the credible region for these two parameters.

It should finally be noticed that the Sorensen model makes use of hyperbolic tangent (tanh) functions, which are numerically effective, but which could be replaced by appropriate Hill functions, more immediately understandable by physiologists and clinicians.

In conclusion, in spite of its limits, the Sorensen model appears to be a flexible and well studied model, able to reproduce realistic physiological behavior from different experiments, particularly in the improved version discussed here. It is hoped that it will be found a useful instrument for the simulation of glucose/insulin system of virtual patients.

## Appendix A1: The Sorensen model

### The Sorensen Model

**Mass Balance—Glucose**
  BRAIN $$\begin{array}{c} V_{BV}^{G}\frac{dG_{BV}}{dt}=Q_{B}^{G}\left(G_{H}-G_{BV}\right)-\frac{V_{BI}}{T_{B}}\left(G_{BV}-G_{BI}\right) \end{array}$$ (23)
    $$\begin{array}{c} V_{BI}\frac{dG_{BI}}{dt}=\frac{V_{BI}}{T_{B}}\left(G_{BV}-G_{BI}\right)-r_{BGU} \end{array}$$ (24)
  HEART AND LUNGS $$\begin{array}{c} V_{H}^{G}\frac{dG_{H}}{dt}=Q_{B}^{G}G_{BV}+Q_{L}^{G}G_{L}+Q_{K}^{G}G_{K}+Q_{P}^{G}G_{PV}-Q_{H}^{G}G_{H}-r_{RBCU} \end{array}$$ (25)
  GUT $$\begin{array}{c} V_{J}^{G}\frac{dG_{J}}{dt}=Q_{J}^{G}\left(G_{H}-G_{J}\right)-r_{JGU} \end{array}$$ (26)
  LIVER $$\begin{array}{c} V_{L}^{G}\frac{dG_{L}}{dt}=Q_{A}^{G}G_{H}+Q_{J}^{G}G_{J}-Q_{L}^{G}G_{L}+r_{HGP}-r_{HGU} \end{array}$$ (27)
  KIDNEY $$\begin{array}{c} V_{K}^{G}\frac{dG_{K}}{dt}=Q_{K}^{G}\left(G_{H}-G_{K}\right)-r_{KGE} \end{array}$$ (28)
  PERIPHERY $$\begin{array}{c} V_{PV}^{G}\frac{dG_{PV}}{dt}=Q_{P}^{G}\left(G_{H}-G_{PV}\right)-\frac{V_{PI}}{T_{P}^{G}}\left(G_{PV}-G_{PI}\right) \end{array}$$ (29)
    $$\begin{array}{c} V_{PI}\frac{dG_{PI}}{dt}=\frac{V_{PI}}{T_{P}^{G}}\left(G_{PV}-G_{PI}\right)-r_{PGU} \end{array}$$ (30)
**Metabolic Source and Sinks—Glucose**
  $$\begin{array}{c} r_{BGU}=70\frac{mg}{min}\;\left[constant\right] \end{array}$$ (31)
  $$\begin{array}{c} r_{RBCU}=10\frac{mg}{min}\;\left[constant\right] \end{array}$$ (32)
  $$\begin{array}{c} r_{JGU}=20\frac{mg}{min}\;\left[constant\right] \end{array}$$ (33)
  $$\begin{array}{c} r_{PGU}=M_{PGU}^{I}M_{PGU}^{G}r_{PGU}^{B} \end{array}$$ (34)
  $$\begin{array}{c} r_{PGU}^{B}=35\frac{mg}{min} \end{array}$$ (35)
  $$\begin{array}{c} M_{PGU}^{I}=7.03+6.52tanh\left[0.338\left(I_{PI}^{N}-5.82\right)\right] \end{array}$$ (36)
  $$\begin{array}{c} M_{PGU}^{G}=G_{PI}^{N} \end{array}$$ (37)
  $$\begin{array}{c} r_{HGP}=M_{HGP}^{I}M_{HGP}^{\Gamma }M_{HGP}^{G}r_{HGP}^{B} \end{array}$$ (38)
  $$\begin{array}{c} r_{HGP}^{B}=155\frac{mg}{min} \end{array}$$ (39)
  $$\begin{array}{c} \frac{dM_{HGP}^{I}}{dt}=\frac{1}{\tau_{I}}\left[M_{HGP}^{I_{\infty }}-M_{HGP}^{I}\right] \end{array}$$ (40)
  $$\begin{array}{c} \tau_{I}=25min \end{array}$$ (41)
  $$\begin{array}{c} M_{HGP}^{I_{\infty }}=1.21-1.14tanh\left[1.66\left(I_{L}^{N}-0.89\right)\right] \end{array}$$ (42)
  $$\begin{array}{c} M_{HGP}^{\Gamma }=M_{HGP}^{\Gamma_{0}}-f_{2} \end{array}$$ (43)
  $$\begin{array}{c} M_{HGP}^{\Gamma_{0}}=2.7tanh\left[0.39\Gamma^{N}\right] \end{array}$$ (44)
  $$\begin{array}{c} \frac{df_{2}}{dt}=\frac{1}{\tau_{\Gamma }}\left(\frac{M_{HGP}^{\Gamma_{0}}-1}{2}-f_{2}\right) \end{array}$$ (45)
  $$\begin{array}{c} \tau_{\Gamma }=65min \end{array}$$ (46)
  $$\begin{array}{c} M_{HGP}^{G}=1.42-1.41tanh\left[0.62\left(G_{L}^{N}-0.497\right)\right] \end{array}$$ (47)
  $$\begin{array}{c} r_{HGU}=M_{HGU}^{I}M_{HGU}^{G}r_{HGU}^{B} \end{array}$$ (48)
  $$\begin{array}{c} r_{HGU}^{B}=20\frac{mg}{min} \end{array}$$ (49)
  $$\begin{array}{c} \frac{dM_{HGU}^{I}}{dt}=\frac{1}{\tau_{I}}\left[M_{HGU}^{I_{\infty }}-M_{HGU}^{I}\right] \end{array}$$ (50)
  $$\begin{array}{c} M_{HGU}^{I_{\infty }}=2tanh\left[0.55I_{L}^{N}\right] \end{array}$$ (51)
  $$\begin{array}{c} M_{HGU}^{G}=5.66+5.66tanh\left[2.44\left(G_{L}^{N}-1.48\right)\right] \end{array}$$ (52)
  $$\begin{array}{c} r_{KGE}=\{\begin{array}{cc} 71+71tanh[0.011\left(G_{K}-460\right)] & 0<G_{K}<460\frac{mg}{min}\\ -330+0.872G_{K} & G_{K}\geq 460\frac{mg}{min} \end{array} \end{array}$$ (53)
**Mass Balance—Insulin**
  BRAIN $$\begin{array}{c} V_{B}^{I}\frac{dI_{B}}{dt}=Q_{B}^{I}\left(I_{H}-I_{B}\right) \end{array}$$ (54)
  HEART AND LUNGS $$\begin{array}{c} V_{H}^{I}\frac{dI_{H}}{dt}=Q_{B}^{I}I_{B}+Q_{L}^{I}I_{L}+Q_{K}^{I}I_{K}+Q_{P}^{I}I_{PV}-Q_{H}^{I}I_{H} \end{array}$$ (55)
  GUT $$\begin{array}{c} V_{J}^{I}\frac{dI_{J}}{dt}=Q_{J}^{I}\left(I_{H}-I_{J}\right) \end{array}$$ (56)
  LIVER $$\begin{array}{c} V_{L}^{I}\frac{dI_{L}}{dt}=Q_{A}^{I}I_{H}+Q_{J}^{I}I_{J}-Q_{L}^{I}I_{L}+r_{PIR}-r_{LIC} \end{array}$$ (57)
  KIDNEY $$\begin{array}{c} V_{K}^{I}\frac{dI_{K}}{dt}=Q_{K}^{I}\left(I_{H}-I_{K}\right)-r_{KIC} \end{array}$$ (58)
  PERIPHERY $$\begin{array}{c} V_{PV}^{I}\frac{dI_{PV}}{dt}=Q_{P}^{I}\left(I_{H}-I_{PV}\right)-\frac{V_{PI}}{T_{PI}^{I}}\left(I_{PV}-I_{PI}\right) \end{array}$$ (59)
    $$\begin{array}{c} V_{PI}\frac{dI_{PI}}{dt}=\frac{V_{PI}}{T_{P}^{I}}\left(I_{PV}-I_{PI}\right)-r_{PIC} \end{array}$$ (60)
**Metabolic Source and Sinks—Insulin**
  $$\begin{array}{c} r_{LIC}=F_{LIC}[Q_{A}^{I}I_{H}+Q_{J}^{I}I_{J}+r_{PIR} \end{array}$$ (61)
  $$\begin{array}{c} F_{LIC}=0.40 \end{array}$$ (62)
  $$\begin{array}{c} r_{KIC}=F_{KIC}\left[Q_{K}^{I}I_{H}\right] \end{array}$$ (63)
  $$\begin{array}{c} F_{KIC}=0.30 \end{array}$$ (64)
  $$\begin{array}{c} r_{PIC}=\frac{I_{PI}}{\left[\left(\frac{1-F_{PIC}}{F_{PIC}}\right)\left(\frac{1}{Q_{P}^{I}}\right)-\frac{T_{P}^{I}}{V_{PI}}\right]} \end{array}$$ (65)
  $$\begin{array}{c} F_{PIC}=0.15 \end{array}$$ (66)
  $$\begin{array}{c} r_{PIR}=\frac{S(G_{H})}{S(G_{H}^{B})}r_{PIR}^{B} \end{array}$$ (67)
  $$\begin{array}{c} \frac{dP}{dt}=\alpha \left[P_{\infty }-P\right] \end{array}$$ (68)
  $$\begin{array}{c} \frac{dI}{dt}=\beta \left[X-I\right] \end{array}$$ (69)
  $$\begin{array}{c} \frac{dQ}{dt}=K\left(Q_{0}-Q\right)+\gamma P-S \end{array}$$ (70)
  $$\begin{array}{c} S=[M_{1}Y+M_{2}{\left(X-I\right)}^{0^{+}}]Q \end{array}$$ (71)
  $$\begin{array}{c} X=\frac{{\left(G_{H}\right)}^{\beta_{pir1}}}{{\left(\beta_{pir2}\right)}^{\beta_{pir1}}+\beta_{pir3}{\left(G_{H}\right)}^{\beta_{pir4}}} \end{array}$$ (72)
  $$\begin{array}{c} P_{\infty }=Y={\left(X\right)}^{1.11} \end{array}$$ (73)
**Mass Balance—Glucagon**
  $$\begin{array}{c} V^{\Gamma }\frac{d\Gamma }{dt}=r_{P\Gamma R}-r_{P\Gamma C} \end{array}$$ (74)
**Metabolic Source and Sinks—Glucagon**
  $$\begin{array}{c} r_{P\Gamma R}=r_{M\Gamma C}\Gamma \end{array}$$ (75)
  $$\begin{array}{c} r_{M\Gamma C}=9.10\frac{ml}{min} \end{array}$$ (76)
  $$\begin{array}{c} r_{P\Gamma R}=M_{P\Gamma R}^{G}M_{P\Gamma R}^{I}r_{P\Gamma R}^{B} \end{array}$$ (77)
  $$\begin{array}{c} M_{P\Gamma R}^{G}=2.93-2.10tanh\left[4.18\left(G_{H}^{N}-0.61\right)\right] \end{array}$$ (78)
  $$\begin{array}{c} M_{P\Gamma R}^{I}=1.31-0.61tanh\left[1.06\left(I_{H}^{N}-0.47\right)\right] \end{array}$$ (79)
**Parameter values**
  Glucose $$\begin{array}{c} \begin{array}{ccc} V_{BV}^{G}=3.5dl & Q_{B}^{G}=5.9\frac{dl}{min} & T_{B}=2.1min\\ V_{BI}=4.5dl & Q_{B}^{G}=43.7\frac{dl}{min} & T_{P}^{G}=5.0min\\ V_{H}^{G}=13.8dl & Q_{A}^{G}=2.5\frac{dl}{min} & \\ V_{L}^{G}=25.1dl & Q_{L}^{G}=12.6\frac{dl}{min} & \\ V_{G}^{G}=11.2dl & Q_{G}^{G}=10.1\frac{dl}{min} & \\ V_{K}^{G}=6.6dl & Q_{K}^{G}=10.1\frac{dl}{min} & \\ V_{PV}^{G}=10.4dl & Q_{P}^{G}V=15.1\frac{dl}{min} & \\ V_{PI}=67.4dl & & \end{array} \end{array}$$
  Insulin $$\begin{array}{c} \begin{array}{ccc} V_{B}^{I}=0.26l & Q_{B}^{I}=0.45\frac{l}{min} & T_{P}^{I}=20min\\ V_{H}^{I}=0.99l & Q_{H}^{I}=3.12\frac{l}{min} & \beta_{pir1}=3.27\\ V_{G}^{I}=0.94l & Q_{A}^{I}=0.18\frac{l}{min} & \beta_{pir2}=132\frac{mg}{dl}\\ V_{L}^{I}=1.14l & Q_{K}^{I}=0.72\frac{l}{min} & \beta_{pir3}=5.93\\ V_{K}^{I}=0.51l & Q_{P}^{I}=1.05\frac{l}{min} & \beta_{pir4}=3.02\\ V_{PV}^{I}=0.74l & Q_{G}^{I}=0.72\frac{l}{min} & \beta_{pir5}=1.11\\ V_{PI}^{I}=6.74l & Q_{L}^{I}=0.90\frac{l}{min} & \\ V_{PI}=6.74l & & \\ M_{1}=0.00747min^{-1} & M_{2}=0.0958min^{-1} & Q_{0}=6.33U\\ \alpha =0.0482min^{-1} & \beta =0.931min^{-1} & K=0.575\frac{U}{min} \end{array} \end{array}$$
  Glucagon $$\begin{array}{c} V^{\Gamma }=11310ml \end{array}$$
**Initial Conditions—Glucose**
  Mass Balance $$\begin{array}{c} G_{PV}^{B}=\left[input\;glucose\;concentration\right] \end{array}$$ (80)
    $$\begin{array}{c} G_{H}^{B}=G_{PV}^{B}+\frac{r_{PGU}^{B}}{G_{P}^{G}} \end{array}$$ (81)
    $$\begin{array}{c} G_{K}^{B}=G_{H}^{B} \end{array}$$ (82)
    $$\begin{array}{c} G_{BV}^{B}=G_{H}^{B}-\frac{r_{BGU}}{G_{B}^{G}} \end{array}$$ (83)
    $$\begin{array}{c} G_{G}^{B}=G_{H}^{B}-\frac{r_{GGU}^{B}}{G_{G}^{G}} \end{array}$$ (84)
    $$\begin{array}{c} G_{L}^{B}=\frac{1}{G_{L}^{B}}\left(Q_{A}^{G}G_{H}^{B}+Q_{G}^{G}G_{G}^{B}+r_{HGP}^{B}-r_{HGU}^{B}\right) \end{array}$$ (85)
    $$\begin{array}{c} G_{BI}^{B}=G_{BV}^{B}-\frac{r_{BGU}T_{B}}{V_{B}I} \end{array}$$ (86)
    $$\begin{array}{c} G_{PI}^{B}=G_{PV}^{B}-\frac{r_{PGU}^{B}T_{P}^{G}}{V_{PI}} \end{array}$$ (87)
  Metabolism $$\begin{array}{c} M_{HGP}^{I}=1 \end{array}$$ (88)
    $$\begin{array}{c} M_{HGU}^{I}=1 \end{array}$$ (89)
    $$\begin{array}{c} f_{2}=0 \end{array}$$ (90)
**Initial Conditions—Insulin**
  Mass Balance $$\begin{array}{c} I_{PV}^{B}=\left[input\;insulin\;concentration\right] \end{array}$$ (91)
    $$\begin{array}{c} I_{H}^{B}=\frac{I_{PV}^{B}}{1-F_{PIC}} \end{array}$$ (92)
    $$\begin{array}{c} I_{K}^{B}=I_{H}^{B}\left(1-F_{KIC}\right) \end{array}$$ (93)
    $$\begin{array}{c} I_{B}^{B}=I_{H}^{B} \end{array}$$ (94)
    $$\begin{array}{c} I_{G}^{B}=I_{H}^{B} \end{array}$$ (95)
    $$\begin{array}{c} I_{PI}^{B}=I_{PV}^{B}-\left[\frac{Q_{P}^{I}T_{P}^{I}}{V_{PI}}\left(I_{H}^{B}-I_{PV}^{B}\right)\right] \end{array}$$ (96)
    $$\begin{array}{c} I_{L}^{B}=\frac{1}{Q_{L}^{I}}\left(Q_{H}^{I}I_{H}^{B}+Q_{B}^{I}I_{B}^{B}+Q_{K}^{I}I_{K}^{B}+Q_{P}^{I}I_{PV}^{B}\right) \end{array}$$ (97)
    $$\begin{array}{c} r_{PIR}^{B}=\frac{Q_{L}^{I}}{1-F_{LIC}}I_{L}^{B}-Q_{G}^{I}I_{G}^{B}-Q_{A}^{I}I_{H}^{B} \end{array}$$ (98)
  Model Pancreas $$\begin{array}{c} X^{B}=\frac{{\left(G_{H}^{B}\right)}^{\beta_{pir1}}}{{\left(\beta_{pir2}\right)}^{\beta_{pir1}}+\beta_{pir3}{\left(G_{H}^{B}\right)}^{\beta_{pir4}}} \end{array}$$ (99)
    $$\begin{array}{c} P\infty ={\left(X^{B}\right)}^{\beta_{pir5}} \end{array}$$ (100)
    $$\begin{array}{c} Y^{B}={\left(X^{B}\right)}^{\beta_{pir5}} \end{array}$$ (101)
    $$\begin{array}{c} P^{B}=P\infty \end{array}$$ (102)
    $$\begin{array}{c} I^{B}=X^{B} \end{array}$$ (103)
    $$\begin{array}{c} Q^{B}=\frac{HQ_{0}+\gamma P\infty }{H+M_{1}Y^{B}} \end{array}$$ (104)
**Initial Conditions—Glucagon**
  Mass Balance $$\begin{array}{c} \Gamma^{B}=\left[input\;plasma\;glucagon\;concentration\right] \end{array}$$ (105)

## Appendix A2: Gastrointestinal models

### Dalla Man et al. model

One of the most known gastric-intestinal models is presented in the work of Dalla Man et al. [58]. This model is composed of three compartments: stomach liquid phase, stomach solid phase and intestine. Glucose oral assumption, its transit through the stomach and the upper small intestine and its absorption in the bloodstream are represented by means of three differential equations and three algebraic functions. The emptying of the stomach is described by a function that depends on the total amount of glucose in the stomach. The system equations are reported below:

$$\begin{array}{c} \left\{ \begin{array}{c} \frac{dq_{sto1}\left(t\right)}{dt}=-k_{21}q_{sto1}\left(t\right)+D\delta \left(t\right)\\ \frac{dq_{sto2}\left(t\right)}{dt}=-k_{empt}q_{sto2}\left(t\right)+k_{21}q_{sto1}\left(t\right)\\ \frac{dq_{gut}\left(t\right)}{dt}=-k_{abs}q_{gut}\left(t\right)+k_{empt}q_{sto2}\\ Ra\left(t\right)=fk_{abs}q_{gut}\left(t\right)\\ k_{empt}\left(q_{sto}\left(t\right)\right)=k_{min}+\frac{k_{max}-k_{min}}{2}\left\{tanh\left[\alpha \left(q_{sto}\left(t\right)-bD\right)\right]-tanh\left[\beta \left(q_{sto}\left(t\right)-cD\right)\right]+2\right\}\\ q_{sto}\left(t\right)=q_{sto1}\left(t\right)+q_{sto2}\left(t\right) \end{array}\right. \end{array}$$ (106)

where:

- *q*_*sto*1_(*t*) and *q*_*sto*2_(*t*) are the amounts of glucose in the stomach (solid phase and liquid phase, respectively);
- *δ*(*t*) is the impulse function;
- *D* is the amount of ingested glucose;
- *q*_*gut*_ is the glucose mass in the intestine;
- *k*_21_ is the rate of grinding;
- *k*_*empt*_ is the rate of gastric emptying;
- *k*_*abs*_ is the rate constant of glucose-intestinal absorption;
- *f* is the fraction of glucose-intestinal absorption which appears in plasma;
- *k*_*max*_ is the maximum value for *k*_*empt*_;
- *k*_*min*_ is the minimum value for *k*_*empt*_;
- *α* and *β* are the rates of decrease and increase, respectively, for the *k*_*empt*_ function;

### Elashoff model

The Elashoff model [59] assumed that the fraction of glucose in the duodenum compartment increases following a power exponential function. The equation system is as it follows:

$$\begin{array}{c} \left\{ \begin{array}{c} \frac{dq_{duo}\left(t\right)}{dt}=D\beta k^{\beta }t^{\beta -1}e^{-{\left(kt\right)}^{\beta }}\\ q_{duo}\left(t\right)=D\left[1-e^{-{\left(kt\right)}^{\beta }}\right] \end{array}\right. \end{array}$$ (107)

where:

- *q*_*duo*_(*t*) is the glucose mass in the duodenum;
- *D* is the amount of ingested glucose;
- *q*_*gut*_ is the glucose mass in the intestine;
- *k* is the rate of emptying;
- *β* is the shape factor.

### Salinari et al. model

In the model reported in Salinari et al. [44], the transit of glucose through the intestine is represented by a mono-dimensional process where the glucose particles are transported from the proximal region to the distal region with a constant velocity. The system equation is the following:

$$\begin{array}{c} \begin{array}{ccc} \frac{\partial q}{\partial t}+u\frac{\partial q}{\partial z}=-\gamma q & z\geq 0 & t\geq 0 \end{array} \end{array}$$ (108)

where:

- *q* is the glucose density;
- *γ* is the rate of glucose absorption.

The gastric emptying is given by the boundary condition in *z* = 0:

$$\begin{array}{c} q\left(0,t\right)=\{\begin{array}{cc} \frac{1}{u}\eta \left(t\right) & 0\leq t\leq 0\\ 0 & t\geq 0 \end{array} \end{array}$$ (109)

where:

- $\eta (t)=D\beta k^{\beta }t^{\beta -1}e^{-{\left(kt\right)}^{\beta }}$ is the rate of glucose delivery in the duodenum;
- *D* is the glucose dose;
- *u* is the constant velocity with which the glucose particles are transported from the proximal region to the distal region.

### Lehmann & Deutsch model

The Lehmann & Deutsch model [60] describes glucose absorption by the gut, assuming that the gastric emptying process is represented by a trapezoidal function. The absorption in the intestine follows a first order kinetics. The system equations are:

$$\begin{array}{c} \left\{ \begin{array}{c} \frac{dq_{gut}\left(t\right)}{dt}=-k_{abs}q_{gut}\left(t\right)+G_{empt}\left(t\right)\\ Ra\left(t\right)=fk_{abs}q_{gut}\left(t\right) \end{array}\right. \end{array}$$ (110)

where

- *q*_*gut*_(*t*) is the amount of glucose in the gut;
- *k*_*abs*_ is the rate constant of glucose intestinal absorption;
- *f* is the fraction of the glucose intestinal absorption which appears in plasma;
- *Ra* is the rate of glucose absorption.

## References

[1] Orasanu G, Plutzky J. The pathologic continuum of diabetic vascular disease. J Am Coll Cardiol. 2009;53:S35–42. 10.1016/j.jacc.2008.09.055 19179216 PMC2663393

[2] Gross JL, de Azevedo MJ, P SS, Canani LH, Caramori ML, Zelmanovitz T. Diabetic nephropathy: diagnosis, prevention, and treatment. Diabetes Care. 2005;28:164–76. 10.2337/diacare.28.1.164 15616252

[3] Solomon SD, Chew E, Duh EJ, Sobrin L, Sun JK, VanderBeek BL, et al Diabetic retinopathy: a position statement by the American Diabetes Association. Diabetes Care. 2017;40:412–18. 10.2337/dc16-2641 28223445 PMC5402875

[4] Pop-Busui R, Boulton AJM, Feldman EL, Bril V, Freeman R, Malik R, et al Diabetic neuropathy: a position statement by the American Diabetes Association. Diabetes Care. 2017;40(1):136–54. 10.2337/dc16-2042 27999003 PMC6977405

[5] Duca L, Sippl R, Snell-Bergeon JK. Is the risk and nature of CVD the same in Type 1 and Type 2 diabetes? Curr Diab Rep. 2013;13:350–61. 10.1007/s11892-013-0380-1 23519720 PMC8986271

[6] Forbes JM, Cooper ME. Mechanisms of diabetic complications. Physiol Rev. 2013;93:137–88. 10.1152/physrev.00045.2011 23303908

[7] Cobelli C, Kovatchev B. Artificial pancreas: past, present, future. Diabetes. 2011;60:2672–2682. 10.2337/db11-0654 22025773 PMC3198099

[8] Hovorka R. Closed-loop insulin delivery from bench to clinical practice. Nature Reviews Endocrinology. 2011;7:385–395. 10.1038/nrendo.2011.32 21343892

[9] Kovacs L, Szalay P, Almassy Z, Barkai L. Applicability Results of a Nonlinear Model-Based Robust Blood Glucose Control Algorithm. Journal of Diabetes Science and Technology. 2013;7:708–716. 10.1177/193229681300700316 23759404 PMC3869139

[10] Palumbo P, Pizzichelli G, Panunzi S, Pepe P, De Gaetano A. Model-based control of plasma glycemia: test on populations of virtual patients. Mathematical Biosciences. 2014;257:2–10. 10.1016/j.mbs.2014.09.003 25223234

[11] Borri A, Cacace F, De Gaetano A, Germani A, Manes C, Palumbo P, et al Luenberger-like observers for nonlinear time-delay systems with application to the artificial pancreas: The attainment of good performance. IEEE Control Systems Magazine. 2017;37(4):33–49. 10.1109/MCS.2017.2696759

[12] Borri A, Palumbo P, Manes C, Panunzi S, De Gaetano A. Sampled-data observer-based glucose control for the artificial pancreas. Acta Polytech Hungarica. 2017;14(1):79–94.

[13] Bergman R, Ider Y, Bowden C, Cobelli C. Quantitative estimation of insulin sensitivity. American Journal of Physiology. 1979;236:667–677. 10.1152/ajpendo.1979.236.6.E667 443421

[14] Panunzi S, Palumbo P, De Gaetano A. A discrete single delay model for the intra-venous glucose tolerance test. Theoretical Biology and Medical Modelling. 2007;4:1–16. 10.1186/1742-4682-4-35 17850652 PMC2072949

[15] Panunzi S, De Gaetano A, Mingrone G. Advantages of the single delay model for the assessment of insulin sensitivity from the intravenous glucose tolerance test. Theoretical Biology and Medical Modelling. 2010;7:9 10.1186/1742-4682-7-9 20298586 PMC2858103

[16] De Gaetano A, Arino O. Mathematical modelling of the intravenous glucose tolerance test. Journal of Mathematical Biology. 2000;40:136–168. 10.1007/s002850050007 10743599

[17] Dalla Man C, Rizza A, Cobelli C. Meal Simulation Model of the Glucose-Insulin System. IEEE Transactions on Biomedical Engineering. 2007;54(10):1740–1749. 10.1109/TBME.2007.893506 17926672

[18] Kovatchev B, Breton M, Dalla Man C, Cobelli C. In silico preclinical trials: a proof of concept in closed-loop control of type 1 diabetes. Journal Diabetes Science Technology. 2009;3:44–55. 10.1177/193229680900300106 PMC2681269 19444330

[19] Dalla Man C, Micheletto F, Lv D, Breton M, Kovatchev B, Cobelli C. The UVA/Padova Type 1 Diabetes Simulator: New Features. journal of Diabetes Science and Technology. 2014;8:26–34. 10.1177/1932296813514502 24876534 PMC4454102

[20] Visentin R, Campos-Náñez E, Schiavon M, Lv D, Vettoretti M, Breton M, et al The UVA/Padova Type I Diabetes Simulator Goes From Single Meal to Single Day. Journal of Diabetes Science and Technology. 2018;12:273–281. 10.1177/1932296818757747 29451021 PMC5851236

[21] Hovorka R, Shojaee-Moradie F, Caroll P, Chassin L, Gowrie I, Jackson N, et al Partitioning glucose dristribution/transport, disposal, and endogenous production during IVGTT. Americal Journal of Physiology. 2002;282:992–1007. 10.1152/ajpendo.00304.2001 11934663

[22] Hovorka R, Canonico V, Chassin L, Haueter U, Massi-Benedetti M, Federici M, et al Nonlinear model predictive control of glucose concentration in subjects with type 1 diabetes. Physiological Measurement. 2004;25:905–920. 10.1088/0967-3334/25/4/010 15382830

[23] Sorensen JT. A Physiologic Model of Glucose Metabolism in Man and Its Use to Design and Improved Insulin Therapies for Diabetes; 1978.

[24] Parker RS, Doyle FJ III, Peppas NA. A Model-Based Algorithm for Blood Glucose Control in Type I Diabetic Patients. Biomedical Engineering. 1999;46(2):148–157. 10.1109/10.740877 9932336

[25] Abate A, Tiwari A, Sastry S. Box invariance in biologically-inspired dynamical systems. Automatica. 2009;45:1601–1610. 10.1016/j.automatica.2009.02.028

[26] Chee F, Fernando T. Closed-Loop Control of Blood Glucose. Springer; 2007. 10.1177/0310057X0203000306 12075636

[27] Steil GM, Rebrin K. Closed-loop insulin delivery—what lies between where we are and where we are going? Asheley Publications. 2005;2:353–362. 10.1517/17425247.2.2.353 16296759

[28] Galwani S, Tiwari A. Constraint-based Approach for Analysis of Hybrid Systems. In: Gupta A., Malik S. (eds) Computer Aided Verification. CAV 2008. Lecture Notes in Computer Science. vol. 5123. Springer, Berlin, Heidelberg; 2008. p. 190–203.

[29] Campos-Delgado DU, Hernandez-Ordoñez M, Fermat R, Gordillo-Moscoso A. Fuzzy-Based Controller for Glucose Regulation in Type-1 Diabetic Patients by Subcutaneous Route. Biomedical Engineering. 2006;53(11):2201–2210. 10.1109/TBME.2006.879461 17073325

[30] Gillis R, Palerm CC, Zisser H, Jovanocič L, Seborg DE, Doyle FJ III. Glucose Estimation and Prediction through Meal Responses Using Ambulatory Subject Data for Advisory Mode Model Predictive Control. Journal of Diabetes Science and Technology. 2007;1:825–833. 10.1177/193229680700100605 19885154 PMC2769674

[31] Kovács L, Benyó B, Bokor J, Benyó Z. Induced *L*_2_-norm minimization of glucose-insulin system for Typed I diabetic patients. Computer Methods and Programs in Biomedicine. 2011;102:105–118. 10.1016/j.cmpb.2010.06.019 20674065

[32] Cameron BD, Baba JS, Coté GL. Measurement of the Glucose Transport Time Dealy Between the Blood and Aqueus Humor of the Eye for the Eventual Development of Noninvasive Glucose Sensor. Diabetes Technology & Therapeutics. 2007;3(2):201–207. 10.1089/152091501300209552 11478325

[33] Markakis MG, Georgios DM, Papavassilopoulos GP, Marmarelis VZ. Model Predictive Control of Blood Glucose in Typer 1 Diabetes: the Principal Dynamic Modes approach. In: 2008 30th Annual International Conference of the IEEE Engineering in Medicine and Biology Society; 2008. p. 5466–5469. 10.1109/IEMBS.2008.4650451 19163954

[34] Galvanin F, Barolo M, Macchietto S, Bezzo F. Optimal Design of Clinical Tests for the Identification of Physiological Models of Type 1 Diabetes Mellitus. Ind Eng Chem Res. 2009;48:1989–2002. 10.1021/ie801209g 21116725

[35] Parker RS, Doyle FJ III, Ward JH, Peppas NA. Robust *H*_∞_ Glucose Control in Diabetes Using a Physiological Model. Bioengineering, Food, And Natural PRoducts. 2000;46(12):2537–2549.

[36] Owens C, Zisser H, Jovanovic L, Srinivasan B, Bonvin D, Doyle FJ III. Run-to-Run Control of Blood Glucose Concentrations for People With Type 1 Diabetes Mellitus. Biomedical Engineering. 2006;53(12):996–1005. 10.1109/TBME.2006.872818 16761826

[37] Parker RS, Doyle FJ III, Peppas NA. The Intravenous Route to Blood Glucose Control. Engineering and Biology. 2001;20:65–73. 10.1109/51.897829 11211662

[38] Saleem MU, Ferman M, Meraj MA. Stability Analysis of Sorensen’s Model for Controllability and Observability. Proceedings of the Pakistan Academy of Sciences: B Life and Environmental Sciences. 2017;54(2):133–145.

[39] Ramprasad Y, Rangaiah GP, Lakshminarayanan S. Robust PID Controller for Blood Glucose Regulation in Type I Diabetics. Ind Eng Chem Res. 2004;43:8257–8268. 10.1021/ie049546a

[40] De Gaetano A, Panunzi S, Matone A, Samson A, Vrbikova J, Bendlova B, et al Routine OGTT: A Robust Model Including Incretin Effect for Precise Identification of Insulin Sensitivity and Secretion in a Single Individual. Plos One. 2013;8:1–16. 10.1371/journal.pone.0070875 PMC3756988 24009656

[41] MATLAB. version 7 (R2009b). Natick, Massachusetts: The MathWorks Inc.; 2009b.

[42] R Core Team. R: A Language and Environment for Statistical Computing; 2018. Available from: http://www.R-project.org/.

[43] Johnson B. Professional Visual Studio 2017 (English Edition). Wrox; 2017.

[44] Salinari S, Bertuzzi A, Mingrone G. Intestinal transit of a glucose bolus and incretin kinetics: a mathematical model with application to the oral glucose tolerance test. Am J Physiol Endocrinol Metab. 2011;300:955–965. 10.1152/ajpendo.00451.2010 21364121

[45] Smith AFM, Roberts GO. Bayesian computation via the Gibbs sampler and related Markov chain Monte Carlo methods. Journal of the Royal Statistical Society. 1993;55:3–23.

[46] Gelfand AE, Hills SE, Racine-Poon A, Smith AFM. Illustration of Bayesian inference in normal data models using Gibbs sampling. Journal of the American Statistical Association. 1990;85:972–985. 10.1080/01621459.1990.10474968

[47] Gelfand AE, Smith AFM. Sampling-based approaches to calculating marginal densities. Journal of the American Statistical Association. 1990;85:398–409. 10.1080/01621459.1990.10476213

[48] Wakefield JC, Smith AFM, Racine-Poon A, Gelfand AE. Bayesian analysis of linear and nonlinear population models using the Gibbs sampler. Applied Statistics. 1994;43:201–222. 10.2307/2986121

[49] Wakefield JC. The Bayesian analysis of population pharmacokinetic models. Journal of the American Statistical Association. 1996;91:62–75. 10.1080/01621459.1996.10476961

[50] Saleem MU, Farman M, Meraj M. A linear control of Hovorka model. SciInt(Lahore). 2016;1:15–18.

[51] Ali S, Nurul NSBF, Md Som A, Mohd Yusof NF. Meal Disturbance Effect on Blood Glucose Control for Type 1 Diabetes Using Improved Hovorka Equations. Key Engineering Materials. 2019;797:158–167. 10.4028/www.scientific.net/KEM.797.158

[52] Wilinska M, Budiman E, Taub M, Elleri D, Allen J, Acerini C, et al Overnight Closed-Loop Insulin Delivery with Model Predictive Control: Assessment of Hypoglycemia and Hyperglycemia Risk Using Simulation Studies. Journal of diabetes science and technology. 2009;3:1109–20. 10.1177/193229680900300514 20144424 PMC2769888

[53] Creutzfeldt W. The incretin concept today. Diabetologia. 1979;16(2):75–85. 10.1007/BF01225454 32119

[54] Creutzfeldt W, Nauck M. Gut hormones and diabetes mellitus. Diabetes/Metabolism Reviews. 1992;8(2):149–177. 10.1002/dmr.5610080206 1425125

[55] Nauck MA, Bartels E, Ørskov C, Ebert R, Creutzfeldt W. Additive insulinotropic effects of exogenous synthetic human gastric inhibitory polypeptide and glucagon-like peptide-1-(7-36) amide infused at near-physiological insulinotropic hormone and glucose concentrations. Journal of Clinical Endocrinology and Metabolism. 1993;76(4):912–917. 10.1210/jcem.76.4.8473405 8473405

[56] Kreymann B, Ghatei MA, Williams G, Bloom SR. GLUCAGON-LIKE PEPTIDE-1 7-36: A PHYSIOLOGICAL INCRETIN IN MAN. The Lancet. 1987;330(8571):1300–1304. 10.1016/S0140-6736(87)91194-9 2890903

[57] Nauck MA, Homberger E, Siegel EG, Allen RC, Eaton RP, Ebert R, et al Incretin effects of increasing glucose loads in man calculated from venous insulin and C-peptide responses. Journal of Clinical Endocrinology and Metabolism. 1986;63(2):492–498. 10.1210/jcem-63-2-492 3522621

[58] Dalla Man C, Camilleri M, Cobelli C. A System Model of Oral Glucose Absorption: Validation on Gold Standard Data. IEEE Transactions on Biomedical Engineering. 2006;53(12):1–7. 10.1109/TBME.2006.883792 17153204

[59] Elashoff JD, Reedy TJ, Meyer JH. Analysis of gastric emptying data. Gastroenterology. 1982;83:1306–1312. 10.1016/S0016-5085(82)80145-5 7129034

[60] Lehmann ED, Deutsh T. A physiological model of glucoseinsulin interaction in type 1 diabetes mellitus. J Biomed Eng. 1992;14:235–242. 10.1016/0141-5425(92)90058-S 1588781

